# Bacterial Leaf Symbiosis in Angiosperms: Host Specificity without Co-Speciation

**DOI:** 10.1371/journal.pone.0024430

**Published:** 2011-09-07

**Authors:** Benny Lemaire, Peter Vandamme, Vincent Merckx, Erik Smets, Steven Dessein

**Affiliations:** 1 Laboratory of Plant Systematics, K.U.Leuven, Leuven, Belgium; 2 Laboratory of Microbiology, Ghent University, Gent, Belgium; 3 Netherlands Centre for Biodiversity Naturalis, Leiden, The Netherlands; 4 National Herbarium of the Netherlands, Leiden University, Leiden, The Netherlands; 5 National Botanic Garden of Belgium, Meise, Belgium; Charité-University Medicine Berlin, Germany

## Abstract

Bacterial leaf symbiosis is a unique and intimate interaction between bacteria and flowering plants, in which endosymbionts are organized in specialized leaf structures. Previously, bacterial leaf symbiosis has been described as a cyclic and obligate interaction in which the endosymbionts are vertically transmitted between plant generations and lack autonomous growth. Theoretically this allows for co-speciation between leaf nodulated plants and their endosymbionts. We sequenced the nodulated *Burkholderia* endosymbionts of 54 plant species from known leaf nodulated angiosperm genera, i.e. *Ardisia*, *Pavetta*, *Psychotria* and *Sericanthe*. Phylogenetic reconstruction of bacterial leaf symbionts and closely related free-living bacteria indicates the occurrence of multiple horizontal transfers of bacteria from the environment to leaf nodulated plant species. This rejects the hypothesis of a long co-speciation process between the bacterial endosymbionts and their host plants. Our results indicate a recent evolutionary process towards a stable and host specific interaction confirming the proposed maternal transmission mode of the endosymbionts through the seeds. Divergence estimates provide evidence for a relatively recent origin of bacterial leaf symbiosis, dating back to the Miocene (5–23 Mya). This geological epoch was characterized by cool and arid conditions, which may have triggered the origin of bacterial leaf symbiosis.

## Introduction

A remarkable diversity of prokaryote-eukaryote symbioses has been described across many taxa and the degree of interaction can vary from loose and temporary associations to highly specific and permanent assemblages [1]. In many associations the prokaryotic symbiont lives within the body of the eukaryote partner, a situation that is known as ‘endosymbiosis’. In this kind of interaction the prokaryote partner is usually referred to as the ‘endosymbiont’, while the eukaryote partner may be considered as the ‘host’. Highly specific microbial endosymbioses have evolved independently many times particularly in insects [2]–[3], sponges [4], nematodes [5] and deep-sea clams [6]. These interactions are considered as obligate because neither the host nor the endosymbiont can survive outside the symbiotic interaction. The obligate endosymbionts are accommodated mostly intracellularly and contribute to the host fitness by provisioning limiting nutrients, whereas the endosymbiont gains a permanent supply of energy-rich carbon compounds from the host [7]. The endosymbionts are primarily vertically transmitted and maintained through host generations, insuring a close and long-term symbiosis [8]–[9]. In plants, however, mutualistic interactions with obligate and vertically inherited symbionts have not been reported so far, except for the *Nostoc*-*Azolla* association [10]–[12] and the bacterial leaf symbiosis [13]. The latter association received little attention but is certainly the most intimate association known between bacteria and higher plants with leaf nodules or galls as a visible morphological aspect of the symbiosis [13].

Bacterial leaf symbiosis or leaf nodulation occurs in about 500 flowering plant species in the families Rubiaceae and Primulaceae. Despite the predominantly pan(sub)tropical distribution range of both families, leaf nodulated plants are restricted to (sub)tropical parts of Africa and Asia [13]. Most of the nodulated species have been reported in the Rubiaceae, more precisely in three distantly related genera *Psychotria* (ca. 80 nodulated species on a total of 1400 species), *Pavetta* (ca. 350 nodulated species on a total of 400 species) and *Sericanthe* (11–12 nodulated species on a total of 17 species). In Primulaceae, 30 nodulated species occur in *Ardisia* (ca. 300 species), two species in *Amblyanthus* (4 species) and three in *Amblyanthopsis* (4 species). However, in *Amblyanthus* and *Amblyanthopsis* the presence of bacterial leaf nodules is uncertain and none of the species have been examined for bacterial endosymbionts [13]. Recently, molecular studies of selected leaf nodulated Rubiaceae and Primulaceae showed that each host plant is associated with a single narrow clade of *Burkholderia* endosymbionts [14]–[17].

The genus *Burkholderia* is known as a versatile group of bacteria, including soil bacteria and plant pathogens, occupying diverse ecological niches [18]. Some *Burkholderia* species are able to establish a close and symbiotic/mutualistic association with other organisms [19]–[20]. Despite numerous efforts to cultivate leaf nodulated bacteria on laboratory media, none of these were successful, suggesting that the endosymbionts need undetermined substances of the host plant (E. Prinsen, University of Antwerp, pers. comm.). As a result, the uncultivable endosymbionts have been named under the *Candidatus* provision for informal naming of species [21]. Furthermore, the plant associated symbionts are known to play a crucial biological role to ascertain survival of the host [22]. Loss of the bacterial partner affects normal growth and development of the host plant, suggesting an altered hormone balance.

Consequently, the presence of obligate and host specific bacteria in leaf nodulated plants supports the idea of a closed symbiotic cycle, as described in many morphological and ontogenetic studies on leaf endosymbioses [13]. Colonies of endosymbionts are permanently maintained in the shoot tip of the host plant so that new developing leaves and flowers are inoculated by the endosymbionts. In a complex sequence of plant-microbe interactions, the endosymbionts are incorporated into the reproductive stages of the host plant and transmitted vertically through the seeds. An obligate, closed and host specific interaction implicates a long-term association between both partners that could be reflected by phylogenetic congruence or co-speciation.

In this study, we focus on the phylogenetic and evolutionary aspects of bacterial leaf symbiosis based on an extensive sampling of nearly 10% of all leaf nodulated plants. We propose to investigate the host specificity and the obligate aspect of the interaction and to test the key hypothesis of an ancient infection within an ancestral leaf nodulated host followed by parallel evolution between both partners.

## Results

### Endosymbiont phylogeny

**Figure 1:**
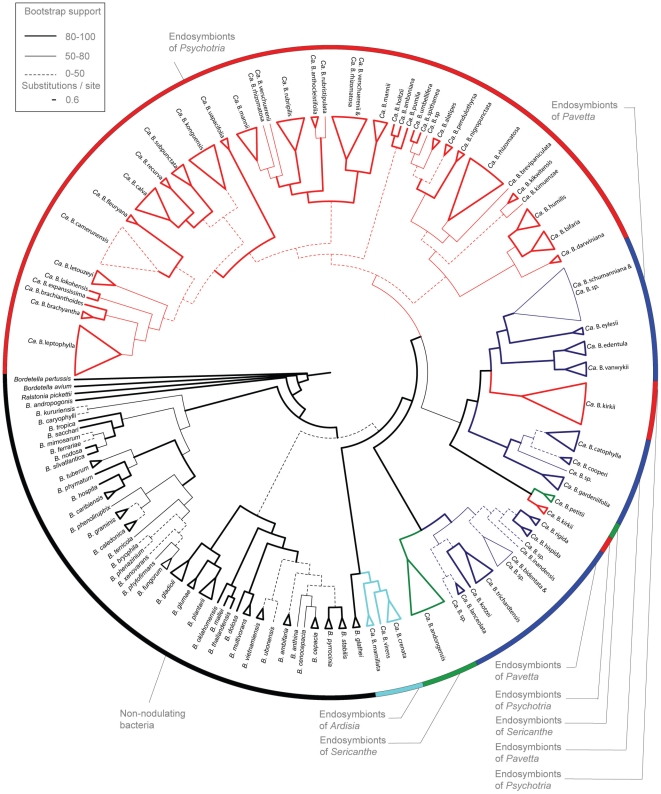
Phylogenetic placement of the *Burkholderia* endosymbionts from leaf nodulated angiosperms. Most optimal Likelihood tree of leaf nodulated angiosperms based on the concatenated alignment of 16S rRNA, *recA* and *gyrB* genes. The leaf nodulated genera are indicated by sections. Maximum Likelihood bootstrap support values (BS) are indicated with thick (80–100 BS), thin (50–100 BS) and dashed (0–50 BS) lines.

From endosymbionts of 14 nodulated *Pavetta* species (representing 45 populations), 35 nodulated *Psychotria* species (representing 107 populations), 2 nodulated *Sericanthe* species (representing 7 populations) and 3 nodulated *Ardisia* species (representing 6 populations), 16S rRNA, *recA* and *gyrB* sequence data were obtained and subjected to molecular phylogenetic analyses together with sequences of non-nodulating *Burkholderia* representatives. Maximum Likelihood and Bayesian analyses of the combined three-gene datasets yielded a robust topology with well-supported relationships between the nodulated species at low and high taxonomical level (Fig. 1; Fig. S1). Several defined and well-supported main clades can be distinguished. The endosymbionts of all nodulated Rubiaceae were recovered as a monophyletic group with high support values (100% Bayesian posterior probability-BPP/99% bootstrap support-BS). The primulaceous endosymbionts were also supported as monophyletic group (100% BPP/100% BS) and placed as sister group with *Burkholderia glathei* (53% BPP/87% BS). The nodulated *Psychotria* endosymbionts form a monophyletic group (100% BPP/46% BS) with exception of the *Psychotria kirkii* endosymbionts. The latter are placed in two different phylogenetic positions: the endosymbionts of seven *Psychotria kirkii* specimens are related with the *Pavetta* endosymbionts (100% BPP/97% BS), while two *Psychotria kirkii* endosymbionts are sister to the endosymbionts of *Sericanthe petitii* (100% BPP/100% BS). The endosymbionts of *Sericanthe andongensis* are related with leaf nodulated *Pavetta* endosymbionts (100% BPP/100% BS) making the endosymbionts of *Sericanthe* biphyletic. The endosymbionts of *Pavetta* are placed in two major clades with complex relationships with *Psychotria* and *Sericanthe* endosymbionts. The first clade contains the endosymbionts of *Pavetta catophylla*, *P. cooperi*, *P. edentula*, *P. eylesii*, *P. gardeniifolia*, *P. schumanniana*, *P. vanwykii*, *Psychotria kirkii*, and *Sericanthe petitii*. The second comprises all remaining endosymbionts of *Pavetta* (*P. bidentata*, *P. hispida*, *P. inandensis*. *P. lanceolata*, *P. kotzei*, and *P. trichardtensis*) as well as the endosymbionts of *Sericanthe andongensis*. Overall, most plant species investigated, except for *Psychotria kirkii*, *P. mannii*, *P. rhizomatosa* and *P. verschuerenii*, were associated with a monophyletic group of *Burkholderia* endosymbionts. In addition, we found no overlap in endosymbionts between the nodulating plant species (Fig. 1; Fig. S1).

**Figure 2:**
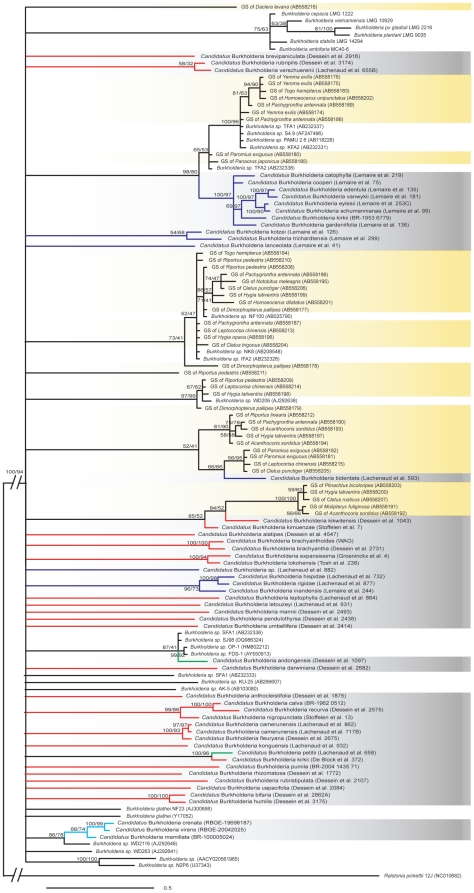
Phylogenetic relationships of leaf nodulated endosymbionts, related insect endosymbionts and free-living *Burkholderia*. Bayesian phylogenetic tree based on 16S rRNA sequences (1208 bp) with support values of Bayesian and Maximum Likelihood analysis (Bayesian posterior probabilities/Maximum Likelihood Bootstrap). Gray and yellow shading denote leaf nodulated and insect gut symbionts, respectively. Voucher information of the leaf nodulated hosts are shown in parentheses. Environmental *Burkholderia* strains with accession numbers are unshaded. Branches of leaf nodulated *Ardisia*, *Pavetta*, *Psychotria* and *Sericanthe* species are indicated in cyan, blue, red and green, respectively. The scale bar represents 0.5 substitutions per site.

Next, our *Burkholderia* 16S rRNA dataset including a comprehensive sample of leaf nodulated endosymbionts and related stinkbug associated symbionts and environmental *Burkholderia* strains was subjected to phylogenetic analysis (Fig. 2; Table S6). The 16S rRNA based tree is more weakly resolved due to a low amount of genetic variability in 16S rRNA but conform to the three-gene phylogeny of leaf nodulated species. Nevertheless, we observed an intermingled phylogenetic pattern between plant, insect and soil bacteria, as previously suggested by Kikuchi et al. [23]. Phylogenetic affinities between soil bacteria and leaf endosymbionts (e.g. *Burkholderia* sp. WD2116 and primulaceous endosymbionts), and between leaf endosymbionts and gut symbionts (e.g. *Candidatus* Burkholderia bidentata and symbionts of Coreid stinkbugs) suggest numerous transmissions of bacteria between different environments (in this case plants, insects and soil).

### Co-speciation

**Figure 3:**
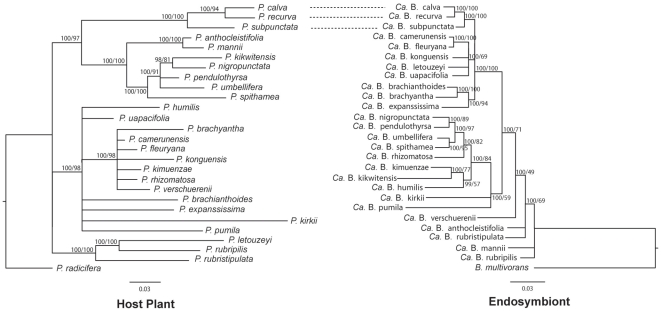
Comparison of host and endosymbiont phylogeny in *Psychotria*. The *Psychotria* host (left) and *Burkholderia* (right) phylograms were constructed from plastid *ndhF-rpl32*, *petD*, *petL-psbE*, *psbD-trnT*, *rps16*, *rps16-trnK*, *trnG*, *trnL-rpl32* and *trnLF* DNA and bacterial 16S rRNA, *recA* and *gyrB* regions, respectively. Bayesian posterior probabilities and Maximum Likelihood bootstrap values are shown above branches. Dashed lines indicate strict co-speciation between host plants and their endosymbionts. Branch lengths represent the number of substitution per site.

The topologies of the Maximum Likelihood and Bayesian analyses of the host and endosymbiont are shown in Fig. 3. Overall, major phylogenetic conflicts at different nodes were observed between the topology of *Psychotria* and their bacterial endosymbionts. Strict and supported topological congruence was only observed between *Psychotria calva*, *P. recurva* and *P. subpunctata* and their associated endosymbionts.

The phylogenetic relationships of the *Psychotria* species and their endosymbionts were compared by TreeMap v.3.0β. For computational reasons of the exhaustive search (1) we omitted four taxa (*Psychotria kirkii*, *P. mannii*, *P. rhizomatosa* and *P. verschuerenii*), known to have host populations with different endosymbiont species (Fig. 1), and (2) we constrained the possibility to lose the essential endosymbionts to zero. The reconciliation analysis of the host ML tree and the endosymbiont tree introduced 12 co-speciation events, 30 duplications, and 15 host switches (total event cost of 35). The randomization test indicated that the results of the reconciliation analysis are statistically significant (P = 0.02+/−0.01) indicating both significant co-speciation and non-co-speciation events between host plants and their endosymbionts.

### Molecular dating

The phylogenetic analysis of the Rubiaceae dataset resulted in a highly resolved consensus tree, showing phylogenetic relationships that are consistent with earlier studies [24]–[25]. We recovered two subfamilies i.e. Rubioideae (BI 100%) and Cinchonoideae (BI 100%), the latter with two supertribes (Ixoridinae BI 100% and Cinchonidinae BI 100%). The phylogenetic relationships within these groups corroborate with detailed studies in Rubioideae [26]–[27], Ixoridinae [28] and Cinchonidinae [29]–[30]. The leaf nodulated genera *Psychotria*, *Pavetta* and *Sericanthe* are recovered as monophyletic groups with maximum branch support. This result is congruent with the studies of Andersson [31], De Block et al. (unpublished) and Davis et al. [32], respectively. The estimated divergence times with credibility intervals obtained for the rubiaceous subfamilies, supertribes and nodulated genera are listed in Table 1. The phylogenetic chronogram of Rubiaceae is shown in Figure S1. The mean ages of the crown group of leaf nodulated *Psychotria*, *Pavetta* and *Sericanthe* lineages are estimated at 9, 4 and 3 Mya, respectively.

**Table 1:** Divergence times of leaf nodulated angiosperms.

Rubiacaee	Group	Stem	Crown
	Rubiaceae	73 (60–86)	62 (50–77)
	Cinchonoideae	62 (55–70)	60 (54–68)
	Cinchonidinae	60 (54–68)	36 (24–52)
	Ixoridinae	60 (54–68)	55 (51–60)
	Rubioideae	62 (55–70)	53 (48–60)
	*Pavetta*	9 (4–15)	4 (2–7)
	Nodulating *Pavetta*	**9 (4–15)**	**4 (2–7)**
	*Sericanthe*	10 (5–16)	5 (2–8)
	Nodulating *Sericanthe*	**5 (2–8)**	**3 (1–5)**
	*Psychotria*	28 (22–34)	19 (14–25)
	Nodulating *Psychotria*	**12 (8–16)**	**9 (6–12)**

asterids	Group	Stem	Crown
	Cornales	137*	106 (92–123)
	Ericales	128 (124–132)	118 (110–125)
	Lamiids	114 (101–125)	102 (87–118)
	Campanulids	114 (101–125)	106 (90–121)
	Aquifoliales	106 (90–121)	84 (52–111)
	Apiales	84 (58–105)	53 (27–81)
	Dispsacales	84 (58–105)	64 (32–90)
	Asterales	89 (68–111)	59 (34–84)
	Garryales	98 (87–118)	63 (31–91)
	Gentianales	76 (56–97)	52 (35–75)
	Solanales	76 (56–97)	71 (50–93)
	Lamiales	81 (61–100)	56 (33–82)
	*Ardisia*	20 (11–29)	10 (5–15)
	Nodulating *Ardisia*	7*	**4 (1–8)**

Estimated time ages in Mya (mean [95%CI]) for the crown and stem groups of the Rubiaceae and asterid plant groups. An asterisk indicates a not supported node without time age confidence interval.

The BEAST analysis (Fig. S2) of the asterid dataset yielded a well-resolved phylogenetic hypothesis congruent with previous investigations [33]–[34]. Our results showed similar phylogenetic uncertainty regarding the interordinal affinities of campanulids (Apiales, Dipsacales and Asterales) and lamiids (Gentianales, Solanales and Lamiales). Within the order Ericales, the Primulaceae were found as a monophyletic group (BI 100%) and placed within the monophyletic primuloid group (i.e. Maesaceae, Theophrastaceae and Primulaceae; e.g. [33], [35]). The genus *Ardisia* is recovered as monophyletic group (BI 100%) and sister to the *Myrsine*-*Rapanea* clade (BI 97%). The interspecific relationships in *Ardisia* received weak support, but the monophyly of leaf nodulating species was strongly supported (BI 100%) as described previously [16], [36]. The divergence time estimations with credibility intervals obtained for the asterids, lamiids, campanulids, Ericales, Cornales, *Ardisia* and the origin of leaf nodulation in *Ardisia* are listed in Table 1. The mean crown-node age of leaf nodulated *Ardisia* species is estimated to 5 Mya. The chronogram of the asterids with calibration points is shown in Figure S2.

## Discussion

### Origin, host-endosymbiont stability and co-speciation

Our results corroborate previous evidence that bacterial leaf nodulation has evolved at least four times independently in angiosperms and has a single origin in the genera *Ardisia* [36], *Pavetta* (De Block et al. unpublished data), *Psychotria* [31], and *Sericanthe* [32]. We detected a genetically closely related *Burkholderia* endosymbiont in the leaf nodules of plant species from each of these four distantly related clades (Fig. 1). This supports previous bacterial identifications in selected nodulated *Psychotria* [14], [37], *Pavetta* [17], *Sericanthe* [15] and *Ardisia* species [16]. Furthermore, our results show that the overwhelming majority of leaf nodulated plant species are consistently associated with a single bacterial partner in a specific manner (except for four species, see below). Different individuals (2 to 8 populations) from different geographical locations (Table S1) were included in the analysis and no overlap of endosymbiont clades was observed between the nodulating plant species. The observation of a highly specific (one-to-one) host-endosymbiont interaction combined with all morphological evidence for a closed cycle (see review of Miller [13]) leads to the proposal of a vertical transmission of the obligate symbionts resulting in a tight long-term co-speciation. However, our phylogenetic analyses reject strict co-speciation and show evidence for an intermittent interaction between plants and their endosymbiont. The endosymbionts of the rubiaceous nodulated genera *Pavetta*, *Psychotria* and *Sericanthe* are not grouped into monophyletic groups, despite the fact that the monophyly of the three distantly related genera is confirmed. For example, the endosymbionts of *Psychotria kirkii*, *Sericanthe andongensis* and *S. petitii* are related to endosymbionts of *Pavetta* species and not to the other endosymbionts of other *Psychotria* and *Sericanthe* species. Although limited, this intermingled phylogenetic pattern among leaf nodulated genera of Rubiaceae indicates multiple evolutionary origins at intergeneric level suggesting horizontally acquired bacteria. In contrast to the Rubiaceae, previous work showed that the endosymbionts of *Ardisia* belong to a distinct clade, sister to the Rubiaceae endosymbionts [16]. This phylogenetic pattern may be an artifact caused by a sampling bias towards Africa and Asia of Rubiaceous and Primulaceous plants, respectively. However, bacteria of Asian nodulated *Pavetta* species (accessions BR-20041440 and BR-20041114076) revealed a close phylogenetic affinity with those of African *Pavetta schumanniana* specimens; this suggests that there is no pronounced geographic differentiation of the endosymbionts in both families.

Additional evidence for host-symbiont mixing was obtained by the co-speciation analysis within nodulated *Psychotria* species. Our observation of significant incongruencies between the phylogenies of the endosymbionts and their *Psychotria* host undermined the idea of strict co-speciation (Fig. 3). However, in few terminal taxa (i.e. common ancestor of *Psychotria calva*, *P. recurva* and *P. subpunctata*), convergence between symbionts and host occurred, showing evidence for an ongoing co-speciation between these taxa. The latter three *Psychotria* species are closely related with few morphologically distinctive characters [38], suggesting recently diversified species.

A possible scenario to explain the observation of host specificity without long-term co-speciation is that the ancestral nodulated Rubiaceae were initially colonized by a broad range of bacterial endosymbionts followed by a recent specialization process of the host plants towards different specific bacterial taxa. This evolutionary shift in specificity has resulted into a specific one-to-one symbiotic interaction. However, the observation of free-living *Burkholderia* nested within the leaf nodulated clades (Fig. 2) suggests frequent reinfection events of leaf nodulated species with soil bacteria. This alternative hypothesis implies an early but diffuse phase of an open bacteria-plant interaction, which allowed multiple external infections from soil bacteria. *Burkholderia* bacteria are commonly isolated from soil environments [39]–[40] and some of them seem to be closely related with gut symbionts of insects (Fig. 2; [23]). Intergeneric and interspecific transmission via these ecological contacts is reasonable to accept and should be further investigated with experimental techniques achieved in the lab. In addition, we found among the host populations of four specimens (i.e. *Psychotria kirkii*, *P. mannii*, *P. rhizomatosa* and *P. verschuerenii*) two or three distinct bacterial lineages, but a stable interaction was demonstrated within different individuals of a given population. These results indicate also recent and ongoing reinfection events at intraspecific level with consistent specificity at population level. A population study comparing fast evolving plant genetic markers and multiple endosymbiont genes is required and might reveal novel information in the transmission mode of endosymbionts in leaf nodulated plants.

### Age of leaf nodulated bacteria-plant associations

The estimated divergence age of the nodulating genera (Table 1) generally corroborates the results of previous studies. Yesson et al. [41] performed a molecular dating analysis of Primulaceae at generic level with a stem node estimate of *Ardisia* at 14–15 Mya. Nie et al. [42] estimated the divergence time of the genus *Kelloggia* within a Rubiaceae wide *rbcL* dataset. In this study the stem node of *Pavetta* was estimated at approximately 13 Mya. In the study of Tosh [43] onset of diversification within the tribe Coffeeae (including *Coffea*, *Sericanthe*, *Tricalysia* in our analysis) occurred during the mid Miocene (approx. 12–16 Mya). For the tribe Psychotrieae (including *Schizocolea*, *Psychotria*, *Morinda*, *Myrmecodia*, *Geophila*, *Chassalia* in our analysis), time estimates range between 35 and 61 Mya [25] and is broadly congruent with the present study. Overall, similarity of age estimates of leaf nodulating groups of published studies and the present study suggests that our molecular dating analysis presents a plausible scenario for the origin and timing of bacterial leaf symbiosis in angiosperms. However, divergence estimates of more basal branches in our analyses, i.e. asterid orders and Rubiaceae subfamilies were more recent compared to the results of Bremer et al. [44] and, Bremer and Eriksson [25], respectively. The largest differences in age estimates were observed for the asterid order Dipsacales and rubiaceous subfamily Cinchonoideae, with more recent stem node ages in this study of 31 and 26 Mya, respectively. Dissimilarities in time estimates are possibly the result of a different molecular dating approach. Using a different inference method, taxon sampling, gene sampling and calibration strategy (e.g. uniform priors vs. lognormal priors for fossil calibration), has been proven to cause differences in age estimates [45]–[46].

The present study suggests that the ancestors of leaf nodulating angiosperms originated in the middle Miocene and continued to diversify throughout the Pliocene (Table 1). In the Miocene (5–23 Mya) climatic conditions changed significantly by global cooling resulting in ice-sheet expansion on Antarctica and the Arctic region [47], and aridification in Asia en Africa [48]–[49]. As a consequence of a drier climate, retraction and impoverishment of rainforest occurred, forcing plant lineages to survive into relatively small but humid refugia [49]–[50]. Under this hypothesis it is reasonable to speculate that climatic change was a possible trigger to promote the bacteria-plant interaction among leaf nodulated species in tropical Africa and Asia. The bacterial nodules of plants might have formed a safe haven for soil bacteria that were confronted with less suitable habitats. On the other hand, uptake of bacteria by the plant might have enhanced plant growth under drought-stress conditions [51]–[53]. This hypothesis might explain why most of the savannah adapted *Psychotria* species are found to be nodulated [38].

## Materials and Methods

### Plant material and taxon sampling

Table S1 lists the leaf nodulating plant species investigated in this study, representing the genera *Pavetta* (14 species), *Sericanthe* (2 species), *Psychotria* (35 species), and *Ardisia* (3 species). Plants were collected from a broad geographic range during different field expeditions to Cameroon, Democratic Republic of the Congo, Gabon, Madagascar, South Africa and Zambia. All collected specimens do not involve endangered species and do not originate from protected localities.

Leaf samples were preserved in silica-gel. Additional nodulated species were obtained from living plants in the National Botanic Garden of Belgium (BR) and the Royal Botanic Garden of Edinburgh (RBGE). Related bacterial sequences of *Burkholderia* were obtained from Genbank. All plants species with voucher and Genbank accession numbers used in the co-speciation and molecular dating analyses are listed in Table S2, Table S3 and Table S4.

### DNA extraction, amplification and sequencing

Bacterial DNA was obtained from excised leaf nodules with the modified CTAB protocol of Tel-Zur et al. [54]. Amplification of bacterial gene sequences (16S rRNA, *recA* and *gyrB*) was carried out as described previously [17]. Plastid DNA regions of plants (*ndhF-rpl32*, *petD*, *petL-psbE*, *psbD-trnT*, *rps16*, *rps16-trnK*, *trnG*, *trnL-rpl32* and *trnLF*) were amplified in a standard 25 µl reaction mix containing 1 µl total DNA, 16 µl H_2_0, 2.5 µl 10× PCR buffer, 0.75 µl 25 µM MgCl_2_, 1 µl of 20 µM forward and reverse primers, 2.5 µl 2 µM dNTP and 0.2 µl Taq DNA polymerase. All DNA amplifications were conducted in a GeneAmp PCR System 9700 (Applied Biosystems, Foster City, California, USA), adopting the temperature profile described by the references listed in Table S5. Amplified products were purified for sequencing by using a modification of the Exo/SAP enzyme cleaning protocol [55]. Purified PCR products were sent to Macrogen for sequencing (Macrogen Inc, Seoul, Korea).

### Datasets

The five datasets employed in this study are described below with taxon and voucher information listed in the supplementary data.

A three-gene dataset with 224 taxa was constructed including the endosymbionts of leaf nodulated angiosperms. The phylogenetic relationships were inferred from 2538 unambiguously aligned nucleotide sites (16S rRNA: 1199 bp, *recA*: 606 bp, and *gyrB*: 733 bp; see Table S1). *Bordetella avium* and *B. pertussis* were used as outgroup.

A 16S rRNA dataset (98 taxa and 1412 bp) was compiled with a selection of our obtained leaf symbiont sequences (52 taxa), supplemented with related *Burkholderia* 16S rRNA accessions (71 taxa) from the study of Kikuchi et al. [23] and Genbank. *Ralstonia picketii* was used as outgroup (Table S6).

To test co-speciation between selected *Psychotria* species and their endosymbionts (27 taxa), a combined host (8232 bp) and endosymbiont (3633 bp) dataset was constructed with respectively nine (*ndhF-rpl32*: 700 bp, *petD*: 1159 bp, *petL-psbE*: 1172 bp, *psbD-trnT*: 1431 bp, *rps16*: 676 bp, *rps16-trnK*: 917 bp, *trnG*: 652 bp, *trnL-rpl32*: 730 bp and *trnLF*: 795 bp) and three (16S rRNA: 1139, *recA*: 589 and *gyrB*: 1905) gene markers. *Psychotria radicifera* and *Burkholderia multivorans* were used as outgroup (Table S2).

To allow inclusion of multiple calibration points for molecular dating analyses of the leaf nodulated angiosperm genera, we constructed 1) a four-gene Rubiaceae dataset of 112 taxa and 5495 bp (*rps16*: 1195 bp, *trnLF*: 1696 bp, *trnG*: 968 bp, *petD*: 1633 bp) and 2) an asterid dataset of 65 taxa and 4572 bp based on *matK* (1977 bp), *rps16* (1117 bp) and *trnLF* (1476 bp) sequences. Selected rubiaceous and primulaceous leaf nodulated lineages (Table S3 and S4) were included in an existing Rubiaceae [56] and asterid [33], [44] dataset.

### Phylogenetic inference

Sequence editing and assembly was done in Geneious Pro v.5.1.7 [57]. Initially, alignments of DNA sequences were generated by using Muscle v.4.0 [58]. In Geneious Pro v. 5.1.7 we manually adjusted the alignment and removed ambiguously aligned regions. Phylogenetic analyses were conducted using Maximum Likelihood (ML) and Bayesian Inference (BI) criteria.

ML analyses were done with RAxML-VI-HPC v2.2.3 [59] using a GTRMIX model of evolution. We performed 100 RAxML runs and selected the topology with the highest likelihood score. Robustness of the ML tree was calculated using a non-parametric bootstrapping on 1000 replicates with GTRMIX set as the nucleotide substitution model. The results of the bootstrap resampling were plotted onto the previously selected ML topology.

BI analyses were carried out using MrBayes v.3.1 [60], with each marker placed in a separate partition and all partitions unlinked. Model selection was done with MrModeltest v. 3.06 [61] under the Akaike information criterion. Four Markov chains (one cold and three heated) were run simultaneously for five million generation, sample frequency and burnin set to 1000 and 2500, respectively. Convergence of the chains was checked using Tracer v.1.4 [62].

### Co-speciation testing

Evidence for congruence of host and endosymbiont phylogeny of Fig. 3 was evaluated with the jungles algorithm implemented in TreeMap v.3.0β [63]. To assess the difference between host and endosymbiont topologies, the fewest possible number of non-co-speciation and maximum number of co-speciation events was estimated under the default settings of the event costs (co-divergence = 0; duplication = host switch = 1). A randomization test of 1000 randomly generated trees was performed to test the null hypothesis that the observed number of co-speciation events was not larger than expected by chance. Completely resolved topologies are necessary for reconciliation analyses in TreeMap. Therefore, the ML trees of hosts and endosymbionts were imported as input trees.

### Molecular dating

A χ^2^-Likelihood-ratio test rejected the hypothesis of clockwise rate of evolution among lineages of our datasets (P<0.05). Therefore, we performed a Bayesian relaxed clock analyses using BEAST 1.6.1 [64] and estimated divergence times on two different datasets. The first analysis (based on *rps16*, *trnLF*, *trnG* and *petD*) was performed with an extensive Rubiaceae dataset representing most Rubiaceae tribes (Table S3) and including the leaf nodulated *Pavetta*, *Psychotria* and *Sericanthe* lineages. The second analysis estimated the origin of nodulated *Ardisia* species in an enlarged *Ardisia*-asterid dataset (based on *matK*, *trnLF* and *rps16*). Sequences covering all major lineages of asterids (Table S4) were available from the study of Bremer et al. [33]. This large-scale approach for both datasets allowed us to integrate multiple fossil calibration points, minimizing bias as a result of a single calibration point.

For both analyses, we applied the GTR+I+G model with 4 gamma categories on each partition. This best fitting model of DNA substitution was chosen by performing hierarchical Likelihood-ratio tests in MrModeltest v. 3.06 [61]. A model of uncorrelated lognormal distributed rates [65] was selected and all fossil calibration points (listed below) were given a lognormal distribution, using the minimum fossil age as lower bound and standard deviation set on 0.5. The distribution of all other priors was set to uniform. BEAST analyses were run using two independent Markov chain Monte Carlo analyses for 30 million generations with sampling every 1000 generations and a burnin of three million generations. Tracer v.1.4 [62] was used to inspect stationarity and convergence of the independent runs and to verify that the effective sampling size exceeded 100.

### Calibration points

To date the asterid tree we used minimum and maximum age constraints for nine different nodes (Fig. S2). Fossil calibration points were adopted from the fossil record of the Asteridae, recently revised by Martinez-Millan [66]: (A) the crown node of the asterids constrained to 128 Mya giving a normal distribution with 128 Mya as mean value and 1 as standard deviation, a calibration point estimated by Bremer et al. [44]; (B) the stem node of the Cornaceae set to 83.5 Mya [67]; (C) the stem node of the Hydrangeaceae set to 89.3 Mya [68]; (D) the crown group of *Diospyros* (Ebenaceae), constrained to 33.9 Mya [69]; (E) the crown group of the Theaceae, constrained to 40.4 Mya [70]; (F) the crown node of the Diapensiaceae set to 83.5 Mya [71]; (G) the crown node of the Actinidiaceae constrained to 70.6 Mya [72]; (H) the crown node of *Pentaphylax* (Pentaphylacaceae) set to 89.3 Mya [73]; and (I) the crown node of the Ericaceae constrained to 89.3 Mya [74].

We used eight calibration points to date the Rubiaceae tree (Fig. S3): (A) the crown node of the Gentianales set to 78 Mya, normally distributed with 78 and 1 as mean value and standard deviation, respectively [44]; (B) the Rubiaceae crown node constrained to 54 Mya, based on the first fossil record of the family [75]. The remaining nodes were calibrated using data from the Rubiaceae fossil pollen record, recently revised by Graham [76]: (C) the crown node of *Chiococca* set to 5.3 Mya; (D) the crown node of *Emmenopterys* constrained to 48 Mya; (E) the crown node of *Ixora* set to 5.3 Mya; (F) the crown node of *Gardenia* constrained to 14.55 Mya; (G) the crown node of *Coprosma* constrained to 23.8 Mya; and (H) the crown node of *Galium* set to 5.3 Mya.

## Supporting Information

Table S1 **Accession numbers, voucher data and origin of** ***Burkholderia*** **strains used in the combined DNA analyses.** Specimens were obtained from the National Botanic Garden of Belgium (BR), the Royal Botanic Garden of Edinburgh (RBGE) and the herbarium of Uppsala (UPS). - = not sequenced.
(PDF)

Table S2 **Accession numbers, voucher data and origin of bacterial endosymbionts and host plants used in the co-speciation analysis.** Specimens were obtained from the National Botanic Garden of Belgium (BR). - = not sequenced.
(PDF)

Table S3 **Accession numbers and voucher data of nodulated genera used in the age estimation analysis of leaf nodulated Rubiaceae.** Specimens were obtained from the National Botanic Garden of Belgium (BR), the Royal Botanic Garden of Edinburgh (RBGE), the Gothenburg herbarium (GB), the herbarium of Leiden (L) and the herbarium of the Missouri Botanical Garden (MO). - = not sequenced.
(PDF)

Table S4 **Accession numbers and voucher data of nodulated** ***Ardisia*** **used in the age estimation analysis of leaf nodulated Primulaceae.** Specimens were obtained from the National Botanic Garden of Belgium (BR) and the Royal Botanic Garden of Edinburgh (RBGE). - = not sequenced.
(PDF)

Table S5 **DNA sequences for primers used in this study.** References (i.e. [17], [77]–[84]) are provided of previously published sequence primers.
(PDF)

Table S6 **16S rRNA accession numbers, voucher data and origin of endosymbionts of leaf nodulated angiosperms, gut symbionts of stinkbugs and environmental isolates.** Specimens were obtained from the National Botanic Garden of Belgium (BR) and the Royal Botanic Garden of Edinburgh (RBGE).
(PDF)

Figure S1 **Phylogenetic relationships within leaf nodulated** ***Burkholderia*** **species based on phylogenetic analysis of 16S rRNA,** ***recA*** **and** ***gyrB*** **data.** Support values of Bayesian and Maximum Likelihood analyses are given at the nodes (Bayesian posterior probabilities - bootstrap values from the Maximum Likelihood analysis). The scale bar represents 0.2 substitutions per site.
(PDF)

Figure S2 **Phylogenetic chronogram of Rubiaceae based on** ***rps16*** **,** ***trnLF*** **,** ***trnG*** **and** ***petD*** **sequence data obtained with a Bayesian relaxed clock analysis.** Bars illustrate the 95% posterior probability intervals on age estimates. Numbers within black boxes indicate calibrated nodes. Yellow shading denotes leaf nodulated lineages. Scale bar below tree measure Mya.
(PDF)

Figure S3 **Phylogenetic chronogram of asterids based on** ***matK*** **,** ***trnLF*** **and** ***rps16*** **sequence data obtained with a Bayesian relaxed clock analysis.** Bars illustrate the 95% posterior probability intervals on age estimates. Numbers within black boxes indicate calibrated nodes. Yellow shading denotes leaf nodulated lineages. Scale bar below tree measure Mya.
(PDF)
