# Supplementary material for: Bacterial Leaf Symbiosis in Angiosperms: Host Specificity without Co-Speciation
Source: PLoS One. 2011 Sep 7;6(9):e24430. doi: 10.1371/journal.pone.0024430 (PMC3168474; doi:10.1371/journal.pone.0024430)
Supplement: Table S1 — Accession numbers, voucher data and origin of Burkholderia strains used in the combined DNA analyses. Specimens were obtained from the National Botanic Garden of Belgium (BR), the Royal Botanic Garden of Edinburgh (RBGE) and the herbarium of Uppsala (UPS). - = not sequenced. (PDF) [file pone.0024430.s001.pdf]

Taxa	Strain/Voucher	Origin	Accession numbers			Host plant
			**16S rDNA**	***recA***	***gyrB***	
Bordetella avium	197N		BAVs02	BAV2309	BAV3410	
Bordetella pertussis	Tohama I		BPr01	BP2546	BP0489	
Ralstonia picketii	12J		NC010678	NC010682	NC010682	
Burkholderia ambifaria	LMG 19182		HQ849072	HQ849130	HQ849186	
Burkholderia ambifaria	MC40-6		NC010552	NC010551	NC010551	
Burkholderia andropogonis	LMG 2129		HQ849073	HQ849131	-	
Burkholderia anthina	LMG 20980		HQ849074	HQ849132	HQ849187	
Burkholderia bryophila	LMG 23644		HQ849075	HQ849133	HQ849188	
Burkholderia caledonica	LMG 19076		AF215704	AY619669	EU024220	
Burkholderia caledonica	LMG 19076		HQ849076	HQ849134	HQ849189	
Burkholderia caribensis	LMG 18531		Y17009	AY644639	GU144374	
Burkholderia caribensis	LMG 18531		HQ849077	HQ849135	HQ849190	
Burkholderia caryophylli	LMG 2155		AB021423	AY619663	AB190573	
Burkholderia cenocepacia	LMG 16656		AF148556	AY951880	DQ124427	
Burkholderia cepacia	LMG 1222		EU024171	AF143786	EU024224	
Burkholderia cepacia	LMG 1222		HQ849078	JF295011	HQ849191	
Burkholderia dolosa	LMG 18943		HQ849079	HQ849136	HQ849192	
Burkholderia ferrariae	LMG 23612		HQ849080	HQ849137	HQ849193	
Burkholderia fungorum	LMG 16225		EU024155	AJ549505	EU024205	
Burkholderia fungorum	LMG 16225		HQ849081	HQ849138	HQ849194	
Burkholderia gladioli	LMG 11626		HQ849082	HQ849139	HQ849195	
Burkholderia gladioli pv. gladioli	LMG 2216		EU024168	AY619665	AB190639	
Burkholderia gladioli pv. gladioli	LMG 2216		HQ849083	HQ849140	HQ849196	
Burkholderia glathei	LMG 14190		U96935	AY619666	EU024198	
Burkholderia glathei	LMG 14190		HQ849084	HQ849141	HQ849197	
Burkholderia glumae	LMG 2196		U96931	AJ551324	EU024213	
Burkholderia glumae	LMG 2196		HQ849085	HQ849142	HQ849198	
Burkholderia graminis	LMG 18924		EU024154	AJ551267	EU024212	
Burkholderia graminis	LMG 18924		HQ849086	HQ849143	HQ849199	
Burkholderia hospita	LMG 20598		HQ849087	HQ849144	HQ849200	
Burkholderia kururiensis	LMG 19447		HQ849088	HQ849145	HQ849201	
Burkholderia mallei	NCTC 10229		NC008835	NC008836	NC008836	

Burkholderia mimosarum	LMG 23256		HQ849089	HQ849146	HQ849202	
Burkholderia multivorans	LMG 13010		HQ849090	-	HQ849203	
Burkholderia multivorans	ATCC 17616		AB092606	AF143775	DQ124425	
Burkholderia nodosa	LMG 23741		HQ849091	HQ849147	HQ849204	
Burkholderia oklahomensis	LMG 23618		HQ849092	HQ849148	HQ849205	
Burkholderia phenazinium	LMG 2247		HQ849093	HQ849149	HQ849206	
Burkholderia phenoliruptrix	LMG 22037		HQ849094	HQ849150	HQ849207	
Burkholderia phymatum	LMG 21445		HQ849095	HQ849151	HQ849208	
Burkholderia phytofirmans	LMG 22487		HQ849096	HQ849152	Bphyt_0003	
Burkholderia plantarii	LMG 9035		U96933	AJ551323	EU024232	
Burkholderia plantarii	LMG 9035		HQ849098	HQ849153	HQ849210	
Burkholderia plantarii	LMG 16020		HQ849097	HQ849154	HQ849209	
Burkholderia pyrrocinia	LMG 14191		U96930	AF143794	EU024236	
Burkholderia pyrrocinia	LMG 14191		HQ849099	HQ849155	HQ849211	
Burkholderia sacchari	LMG 19450		HQ849100	HQ849156	HQ849212	
Burkholderia silvatlantica	LMG 23149		HQ849102	HQ849157	HQ849213	
Burkholderia stabilis	LMG 14294		EU024183	AF456031	DQ124428	
Burkholderia stabilis	LMG 14294		HQ849103	HQ849159	JF295010	
Burkholderia terricola	LMG 20581		HQ849104	HQ849160	HQ849215	
Burkholderia thailandensis	LMG 20219		EF535235	AY619656	EU024228	
Burkholderia tropica	LMG 22274		HQ849105	HQ849161	HQ849216	
Burkholderia tuberum	LMG 21444		EU024149	AY644642	EU024193	
Burkholderia tuberum	LMG 21444		HQ849106	HQ849162	HQ849217	
Burkholderia ubonensis	LMG 20358		EU024179	AY780511	EU024234	
Burkholderia vietnamiensis	LMG 10929		AF097534	AF143793	EU024229	
Burkholderia vietnamiensis	LMG 10929		HQ849107	HQ849163	HQ849218	
Burkholderia xenovorans	LMG 21463		HQ849108	HQ849164	HQ849219	
Candidatus Burkholderia alatipes	BR-Dessein et al. 2547	Cameroon	JN053515	JN054098	JN053967	*Psychotria alatipes* Wernham
Candidatus Burkholderia alatipes	BR-Dessein et al. 2555	Cameroon	JN053516	JN054099	JN053968	*Psychotria alatipes* Wernham
Candidatus Burkholderia amboniana	UPS-Luke 8344	Kenya	-	JN054100	JN053969	*Psychotria amboniana* K.Schum.
Candidatus Burkholderia andongensis	BR-Dessein et al. 1097	Zambia	JF916921	JF916915	JF916905	*Sericanthe andongensis* (Hiern) Robbr.
Candidatus Burkholderia andongensis	BR-Lemaire et al. 259	South Africa	-	JF916912	JF916907	*Sericanthe andongensis* (Hiern) Robbr.
Candidatus Burkholderia andongensis	BR-Lemaire et al. 271	South Africa	JF916918	JF916913	JF916908	*Sericanthe andongensis* (Hiern) Robbr.
Candidatus Burkholderia andongensis	BR-Lemaire et al. 286	South Africa	JF916919	-	JF916906	*Sericanthe andongensis* (Hiern) Robbr.

Candidatus Burkholderia andongensis	BR-Lemaire et al. 293	South Africa	JF916920	JF916914	JF916909	*Sericanthe andongensis* (Hiern) Robbr.
Candidatus Burkholderia anthocleistifolia	BR-Dessein et al. 1875	Gabon	JN053517	JN054101	JN053970	*Psychotria anthocleistifolia* spec. nov.
Candidatus Burkholderia anthocleistifolia	BR-Dessein et al. 1917	Gabon	JN053518	JN054102	JN053971	*Psychotria anthocleistifolia* spec. nov.
Candidatus Burkholderia bidentata	BR-Lachenaud et al. 593	Cameroon	JN053519	JN054103	JN053972	*Pavetta bidentata* Hiern
Candidatus Burkholderia bifaria	BR-Dessein et al. 2862A	Cameroon	JN053520	JN054104	JN053973	*Psychotria bifaria* Hiern
Candidatus Burkholderia bifaria	BR-Dessein et al. 2862D	Cameroon	JN053521	JN054105	JN053974	*Psychotria bifaria* Hiern
Candidatus Burkholderia bifaria	BR-Lachenaud et al. 707	Cameroon	JN053522	JN054106	JN053975	*Psychotria bifaria* Hiern
Candidatus Burkholderia bifaria	BR-Lachenaud et al. 707A	Cameroon	JN053523	JN054107	JN053976	*Psychotria bifaria* Hiern
Candidatus Burkholderia brachyanthoides	BR-2009044596	Unknown	JN053524	JN054108	JN053977	*Psychotria brachyanthoides* De Wild.
Candidatus Burkholderia brachyantha	BR-Dessein et al. 2731	Cameroon	JN053525	JN054109	JN053978	*Psychotria brachyantha* Hiern
Candidatus Burkholderia brachyantha	BR-Lachenaud et al. 876B	Cameroon	JN053526	JN054110	JN053979	*Psychotria brachyantha* Hiern
Candidatus Burkholderia brevipaniculata	BR-Dessein et al. 2916	Cameroon	JN053527	JN054111	JN053980	*Psychotria brevipaniculata* De Wild.
Candidatus Burkholderia calva	BR-19620512	Unknown	HQ849116	HQ849172	JF295009	*Psychotria calva* Hiern
Candidatus Burkholderia calva	BR-19640306	Ivory Coast	HQ849117	HQ849173	HQ849227	*Psychotria calva* Hiern
Candidatus Burkholderia calva	BR-Lachenaud et al. 748A	Cameroon	JN053528	JN054112	JN053981	*Psychotria calva* Hiern
Candidatus Burkholderia calva	BR-Lachenaud et al. 748B	Cameroon	JN053529	JN054113	JN053982	*Psychotria calva* Hiern
Candidatus Burkholderia camerunensis	BR-Dessein et al. 1465	Unknown	JN053530	JN054114	JN053983	*Psychotria camerunensis* E.M.A.Petit
Candidatus Burkholderia camerunensis	BR-Dessein et al. 1390	Cameroon	JN053531	JN054115	JN053984	*Psychotria camerunensis* E.M.A.Petit
Candidatus Burkholderia camerunensis	BR-Dessein et al. 3165A	Cameroon	JN053532	JN054198	JN053986	*Psychotria camerunensis* E.M.A.Petit
Candidatus Burkholderia camerunensis	BR-Dessein et al. 3165B	Cameroon	JN053533	JN054199	JN053987	*Psychotria camerunensis* E.M.A.Petit
Candidatus Burkholderia camerunensis	BR-Lachenaud et al. 717A	Cameroon	JN053534	JN054200	JN053988	*Psychotria camerunensis* E.M.A.Petit
Candidatus Burkholderia camerunensis	BR-Lachenaud et al. 717B	Cameroon	JN053535	JN054201	JN053989	*Psychotria camerunensis* E.M.A.Petit
Candidatus Burkholderia camerunensis	BR-Lachenaud et al. 862	Cameroon	JN053536	JN054116	JN053985	*Psychotria camerunensis* E.M.A.Petit
Candidatus Burkholderia catophylla	BR-Lemaire et al. 179	South Africa	JN053537	JN054117	JN053990	*Pavetta catophylla* K.Schum.
Candidatus Burkholderia catophylla	BR-Lemaire et al. 180	South Africa	JN053538	JN054118	JN053991	*Pavetta catophylla K.Schum.*
Candidatus Burkholderia catophylla	BR-Lemaire et al. 182	South Africa	JN053539	JN054119	-	*Pavetta catophylla K.Schum.*
Candidatus Burkholderia catophylla	BR-Lemaire et al. 219	South Africa	JN053540	JN054120	JN053992	*Pavetta catophylla K.Schum.*
Candidatus Burkholderia rhizomatosa	BR-Dessein et al. 2239	Gabon	JN053595	JN054178	JN054049	*Psychotria cf rhizomatosa* De Wild.
Candidatus Burkholderia cooperi	BR-Lemaire et al. 75	South Africa	JN053541	JN054121	JN053993	*Pavetta cooperi* Harv. & Sond.
Candidatus Burkholderia cooperi	BR-Lemaire et al. 247	South Africa	JN053542	JN054122	JN053994	*Pavetta cooperi* Harv. & Sond.
Candidatus Burkholderia crenata	BR-19073685	Unknown	JF416281	-	JF416287	*Ardisia crenata* Roxb.
Candidatus Burkholderia crenata	BR-19073686	Unknown	JF416282	-	JF416288	*Ardisia crenata* Roxb.
Candidatus Burkholderia crenata	RBGE-19696187	Unknown	JF416283	-	JF416289	*Ardisia crenata* Roxb.
Candidatus Burkholderia darwiniana	BR-Dessein et al. 2682	Cameroon	JN053543	JN054123	JN053995	*Psychotria darwiniana* Cheek

Candidatus Burkholderia darwiniana	BR-Dessein et al. 2720A	Cameroon	JN053544	JN054124	JN053996	*Psychotria darwiniana* Cheek
Candidatus Burkholderia edentula	BR-Lemaire et al. 60	South Africa	JN053545	JN054125	JN053997	*Pavetta edentula* Sond.
Candidatus Burkholderia edentula	BR-Lemaire et al. 70C	South Africa	JN053546	JN054126	JN053998	*Pavetta edentula* Sond.
Candidatus Burkholderia edentula	BR-Lemaire et al. 135	South Africa	JN053547	JN054127	JN053999	*Pavetta edentula* Sond.
Candidatus Burkholderia expanssissima	BR-Groeninckx et al. 4	Madagascar	JN053548	JN054128	JN054000	*Psychotria expansissima* K.Schum.
Candidatus Burkholderia eylesii	BR-Lemaire et al. 87	South Africa	JN053549	JN054129	JN054001	*Pavetta eylesii* S.Moore
Candidatus Burkholderia eylesii	BR-Lemaire et al. 253C	South Africa	JN053550	-	JN054002	*Pavetta eylesii* S.Moore
Candidatus Burkholderia fleuryana	BR-Dessein et al. 2578	Cameroon	JN053551	JN054130	JN054003	*Psychotria fleuryana* E.M.A.Petit
Candidatus Burkholderia fleuryana	BR-Dessein et al. 2675	Cameroon	JN053552	JN054131	JN054004	*Psychotria fleuryana* E.M.A.Petit
Candidatus Burkholderia gardeniifolia	BR-Lemaire et al. 276	South Africa	JN053553	JN054132	JN054005	*Pavetta gardeniifolia* Hochst. ex A.Rich.
Candidatus Burkholderia gardeniifolia	BR-Lemaire et al. 252	South Africa	JN053554	-	JN054006	*Pavetta gardeniifolia* Hochst. ex A.Rich.
Candidatus Burkholderia gardeniifolia	BR-Lemaire et al. 136	South Africa	JN053555	JN054133	JN054007	*Pavetta gardeniifolia* Hochst. ex A.Rich.
Candidatus Burkholderia hispidae	BR-Dessein et al. 3176	Cameroon	HQ849122	HQ849178	HQ849231	*Pavetta hispida* Hiern
Candidatus Burkholderia hispidae	BR-Lachenaud et al. 732	Cameroon	HQ849123	HQ849179	HQ849232	*Pavetta hispida* Hiern
Candidatus Burkholderia holtzii	UPS-Luke 8342	Kenya	-	JN054134	JN054008	*Psychotria holtzii* (K.Schum.) E.M.A.Petit
Candidatus Burkholderia humilis	BR-Dessein et al. 1497	Cameroon	JN053556	JN054136	JN054010	*Psychotria humilis* Hiern
Candidatus Burkholderia humilis	BR-Dessein et al. 1581	Cameroon	-	JN054135	JN054009	*Psychotria humilis* Hiern
Candidatus Burkholderia humilis	BR-Dessein et al. 3175	Cameroon	JN053557	JN054137	JN054011	*Psychotria humilis* Hiern
Candidatus Burkholderia humilis	BR-Lachenaud et al. 820	Cameroon	-	JN054138	JN054012	*Psychotria humilis* Hiern
Candidatus Burkholderia inandensis	BR-Lemaire et al. 244	South Africa	JN053558	JN054139	JN054013	*Pavetta inandensis* Bremek.
Candidatus Burkholderia kikwitensis	BR-2004145187	Zambia	JN053559	JN054140	JN054014	*Psychotria kikwitensis* De Wild.
Candidatus Burkholderia kikwitensis	BR-Dessein et al. 1043	Zambia	JN053560	JN054141	JN054015	*Psychotria kikwitensis* De Wild.
Candidatus Burkholderia kimuenzae	BR-Stoffelen et al. 7	D.R.Congo	JN053561	JN054142	JN054016	*Psychotria kimuenzae* De Wild.
Candidatus Burkholderia kirkii	BR-19536779	Unknown	HQ849109	HQ849165	HQ849220	*Psychotria kirkii* Hiern
Candidatus Burkholderia kirkii	BR-2000194661	D.R.Congo	HQ849110	HQ849166	HQ849221	*Psychotria kirkii* Hiern
Candidatus Burkholderia kirkii	BR-19750521	D.R.Congo	JN053562	JN054143	JN054017	*Psychotria kirkii* Hiern
Candidatus Burkholderia kirkii	BR-2002152647	Unknown	HQ849111	HQ849167	HQ849222	*Psychotria kirkii* Hiern
Candidatus Burkholderia kirkii	BR-2002120315	Unknown	HQ849112	HQ849168	HQ849223	*Psychotria kirkii* Hiern
Candidatus Burkholderia kirkii	BR-2001051392	Unknown	HQ849113	HQ849169	HQ849224	*Psychotria kirkii* Hiern
Candidatus Burkholderia kirkii	BR-1998182519	Kenya	HQ849114	HQ849170	HQ849225	*Psychotria kirkii* Hiern
Candidatus Burkholderia kirkii	BR-200103624	Unknown	HQ849115	HQ849171	HQ849226	*Psychotria kirkii* Hiern
Candidatus Burkholderia konguensis	BR-Dessein et al. 2306	Gabon	JN053563	JN054151	JN054025	*Psychotria konguensis* Hiern
Candidatus Burkholderia konguensis	BR-Dessein et al. 1434	Cameroon	JN053564	JN054149	JN054023	*Psychotria konguensis* Hiern
Candidatus Burkholderia konguensis	BR-Dessein et al. 1705	Gabon	JN053565	JN054150	JN054024	*Psychotria konguensis* Hiern

Candidatus Burkholderia konguensis	BR-Lachenaud et al. 636E	Cameroon	JN053566	JN054152	JN054026	*Psychotria konguensis* Hiern
Candidatus Burkholderia konguensis	BR-Lachenaud et al. 932	Cameroon	JN053567	JN054153	JN054027	*Psychotria konguensis* Hiern
Candidatus Burkholderia kotzei	BR-Lemaire et al. 126	South Africa	JN053568	JN054154	JN054028	*Pavetta kotzei* Bremek.
Candidatus Burkholderia lanceolata	BR-Lemaire et al. 40	South Africa	JN053569	JN054155	JN054029	*Pavetta lanceolata* Eckl.
Candidatus Burkholderia lanceolata	BR-Lemaire et al. 41	South Africa	JN053570	JN054156	JN054030	*Pavetta lanceolata* Eckl.
Candidatus Burkholderia leptophylla	BR-Dessein et al. 3111A	Cameroon	JN053571	JN054157	JN054031	*Psychotria leptophylla* Hiern
Candidatus Burkholderia leptophylla	BR-Dessein et al. 3111B	Cameroon	JN053572	JN054158	JN054032	*Psychotria leptophylla* Hiern
Candidatus Burkholderia leptophylla	BR-Dessein et al. 3159	Cameroon	JN053573	JN054159	JN054033	*Psychotria leptophylla* Hiern
Candidatus Burkholderia leptophylla	BR-Dessein et al. 2570B	Cameroon	JN053574	JN054160	JN054034	*Psychotria leptophylla* Hiern
Candidatus Burkholderia leptophylla	BR-Dessein et al. 2709A	Cameroon	JN053575	JN054161	-	*Psychotria leptophylla* Hiern
Candidatus Burkholderia leptophylla	BR-Dessein et al. 2824A	Cameroon	JN053576	JN054162	JN054035	*Psychotria leptophylla* Hiern
Candidatus Burkholderia leptophylla	BR-Lachenaud et al. 591	Cameroon	JN053577	JN054163	JN054036	*Psychotria leptophylla* Hiern
Candidatus Burkholderia leptophylla	BR-Lachenaud et al. 864	Cameroon	JN053578	JN054164	JN054037	*Psychotria leptophylla* Hiern
Candidatus Burkholderia letouzeyi	BR-Dessein et al. 1731	Gabon	JN053579	JN054165	JN054038	*Psychotria letouzeyi* E.M.A.Petit
Candidatus Burkholderia letouzeyi	BR-Dessein et al. 2140	Gabon	JN053580	JN054166	JN054039	*Psychotria letouzeyi* E.M.A.Petit
Candidatus Burkholderia letouzeyi	BR-Lachenaud et al. 931	Cameroon	JN053581	JN054167	JN054040	*Psychotria letouzeyi* E.M.A.Petit
Candidatus Burkholderia lokohensis	BR-Tosh et al. 238	Madagascar	JN053582	JN054168	JN054041	*Psychotria lokohensis* Bremek.
Candidatus Burkholderia mamillata	BR-10005023	Unknown	JF416284	JN054169	JF416290	*Ardisia mamillata* Hance
Candidatus Burkholderia mamillata	BR-10005024	Unknown	JF416285	JN054170	JF416291	*Ardisia mamillata* Hance
Candidatus Burkholderia mannii	BR-Dessein et al. 2493	Gabon	JN053583	JN054171	JN054042	*Psychotria mannii* Hiern
Candidatus Burkholderia mannii	BR-Dessein et al. 1793	Gabon	JN053584	JN054172	JN054043	*Psychotria mannii* Hiern
Candidatus Burkholderia mannii	BR-Dessein et al. 1807	Gabon	JN053585	JN054173	JN054044	*Psychotria mannii* Hiern
Candidatus Burkholderia mannii	BR-Dessein et al. 2053	Gabon	JN053586	JN054175	JN054045	*Psychotria mannii* Hiern
Candidatus Burkholderia mannii	BR-Dessein et al. 2053	Gabon	-	JN054174	JN054046	*Psychotria mannii* Hiern
Candidatus Burkholderia mannii	BR-Dessein et al. 2299	Gabon	JN053587	JN054176	JN054047	*Psychotria mannii* Hiern
Candidatus Burkholderia mannii	BR-Dessein et al. 2375	Gabon	JN053588	JN054177	JN054048	*Psychotria mannii* Hiern
Candidatus Burkholderia nigropunctata	BR-Dessein et al. 1849	D.R.Congo	HQ849118	HQ849174	HQ849228	*Psychotria nigropunctata* Hiern
Candidatus Burkholderia nigropunctata	BR-Stoffelen et al. 13	Gabon	HQ849119	HQ849175	JF295008	*Psychotria nigropunctata* Hiern
Candidatus Burkholderia pendulothyrsa	BR-Dessein et al. 2438	Gabon	JN053589	JN054179	JN054050	*Psychotria pendulothyrsa* spec. nov.
Candidatus Burkholderia pendulothyrsa	BR-Lachenaud et al. 647B	Cameroon	JN053590	JN054180	JN054051	*Psychotria pendulothyrsa* spec. nov.
Candidatus Burkholderia petitii	BR-Dessein et al. 1592	Cameroon	JF916923	JF916916	JF916911	*Sericanthe petitii* (N.Hallé) Robbr.
Candidatus Burkholderia petitii	BR-Lachenaud et al. 658	Cameroon	JF916922	JF916917	JF916910	*Sericanthe petitii* (N.Hallé) Robbr.
Candidatus Burkholderia pumila	BR-2004143571	Zambia	JN053591	JN054181	JN054052	*Psychotria pumila* Hiern
Candidatus Burkholderia kirkii	BR-De Block et al. 372	Kenya	JN053592	JN054182	JN054053	*Psychotria kirkii* Hiern

Candidatus Burkholderia recurva	BR-Dessein et al. 2550A	Cameroon	JN053593	JN054183	JN054054	*Psychotria recurva* Hiern
Candidatus Burkholderia recurva	BR-Dessein et al. 2575	Cameroon	JN053594	JN054184	JN054055	*Psychotria recurva* Hiern
Candidatus Burkholderia rhizomatosa	BR-Dessein et al. 1772	Gabon	JN053596	JN054185	JN054056	*Psychotria rhizomatosa* De Wild.
Candidatus Burkholderia rhizomatosa	BR-Dessein et al. 1785	Gabon	JN053598	JN054187	JN054057	*Psychotria rhizomatosa* De Wild.
Candidatus Burkholderia rhizomatosa	BR-Dessein et al. 2092	Gabon	JN053599	JN054188	JN054058	*Psychotria rhizomatosa* De Wild.
Candidatus Burkholderia rhizomatosa	BR-Dessein et al. 2223	Gabon	JN053600	JN054189	JN054059	*Psychotria rhizomatosa* De Wild.
Candidatus Burkholderia rhizomatosa	BR-Dessein et al. 2240	Gabon	JN053601	JN054190	JN054060	*Psychotria rhizomatosa* De Wild.
Candidatus Burkholderia rhizomatosa	BR-Dessein et al. 2368	Gabon	JN053597	JN054186	JN054061	*Psychotria rhizomatosa* De Wild.
Candidatus Burkholderia rhizomatosa	BR-Dessein et al. 2432	Gabon	JN053602	JN054191	JN054062	*Psychotria rhizomatosa* De Wild.
Candidatus Burkholderia rhizomatosa	BR-Dessein et al. 2551B	Cameroon	JN053603	JN054192	JN054063	*Psychotria rhizomatosa* De Wild.
Candidatus Burkholderia rhizomatosa	BR-Dessein et al. 2674	Cameroon	JN053604	JN054193	JN054064	*Psychotria rhizomatosa* De Wild.
Candidatus Burkholderia rigidae	BR-Lachenaud et al. 694	Cameroon	HQ849120	HQ849176	HQ849229	*Pavetta rigida* Hiern
Candidatus Burkholderia rigidae	BR-Lachenaud et al. 877	Cameroon	HQ849121	HQ849177	HQ849230	*Pavetta rigida* Hiern
Candidatus Burkholderia rubripilis	BR-Dessein et al. 1806	Gabon	JN053605	-	JN054065	*Psychotria rubripilis* K.Schum.
Candidatus Burkholderia rubripilis	BR-Dessein et al. 2295	Gabon	JN053606	JN054194	JN054066	*Psychotria rubripilis* K.Schum.
Candidatus Burkholderia rubripilis	BR-Dessein et al. 1973	Gabon	JN053607	JN054195	JN054067	*Psychotria rubripilis* K.Schum.
Candidatus Burkholderia rubripilis	BR-Dessein et al. 2077	Gabon	JN053608	JN054196	JN054068	*Psychotria rubripilis* K.Schum.
Candidatus Burkholderia rubripilis	BR-Dessein et al. 3174	Cameroon	JN053609	JN054197	JN054069	*Psychotria rubripilis* K.Schum.
Candidatus Burkholderia rubristipulata	BR-Dessein et al. 2107	Gabon	JN053610	-	JN054070	*Psychotria rubristipulata* R.D.Good
Candidatus Burkholderia schumanniana	BR-Dessein et al. 1137	Zambia	HQ849125	HQ849181	HQ849234	*Pavetta schumanniana* F.Hoffm.ex K.Schum.
Candidatus Burkholderia schumanniana	BR-2001944257	D.R.Congo	HQ849126	HQ849182	HQ849235	*Pavetta schumanniana* F.Hoffm.ex K.Schum.
Candidatus Burkholderia schumanniana	BR-2004143066	Zambia	HQ849127	HQ849183	HQ849236	*Pavetta schumanniana* F.Hoffm.ex K.Schum.
Candidatus Burkholderia schumanniana	BR-Dessein et al. 1099	Zambia	HQ849124	HQ849180	HQ849233	*Pavetta schumanniana* F.Hoffm.ex K.Schum.
Candidatus Burkholderia schumanniana	BR-Lemaire et al. 1	South Africa	HQ849128	HQ849184	HQ849237	*Pavetta schumanniana* F.Hoffm.ex K.Schum.
Candidatus Burkholderia schumanniana	BR-Lemaire et al. 99	South Africa	HQ849129	HQ849185	HQ849238	*Pavetta schumanniana* F.Hoffm.ex K.Schum.
Candidatus Burkholderia sp	BR-20041440	Unknown	JN053611	JN054202	JN054071	*Pavetta sp.* L.
Candidatus Burkholderia sp	BR-2004114076	Unknown	JN053612	JN054204	JN054073	*Pavetta sp.* L.
Candidatus Burkholderia sp	BR-2006012338	Unknown	JN053613	JN054203	JN054072	*Pavetta sp.* L.
Candidatus Burkholderia sp	BR-Dessein et al. 1974	Gabon	JN053614	JN054205	JN054074	*Psychotria sp.* L.
Candidatus Burkholderia sp	BR-Dessein et al. 3171	Cameroon	JN053615	-	JN054075	*Pavetta sp.* L.
Candidatus Burkholderia sp	BR-Dessein et al. 2719	Cameroon	JN053616	JN054206	JN054076	*Pavetta sp.* L.
Candidatus Burkholderia sp	BR-Lachenaud et al. 616	Cameroon	JN053617	JN054207	JN054077	*Pavetta sp.* L.
Candidatus Burkholderia sp	BR-Lachenaud et al. 919	Cameroon	JN053618	JN054208	JN054078	*Pavetta sp.* L.
Candidatus Burkholderia sp.	BR-Lachenaud et al. 882	Cameroon	JN053619	-	-	*Pavetta sp.* L.

Candidatus Burkholderia spithamea	BR-2000194762	D.R.Congo	JN053620	-	JN054079	*Psychotria spithamea* S.Moore
Candidatus Burkholderia subpunctata	BR-Dessein et al. 2475	Gabon	-	JN054209	JN054080	*Psychotria subpunctata* Hiern
Candidatus Burkholderia subpunctata	BR-Lachenaud et al. 775A	Cameroon	JN053621	JN054210	JN054081	*Psychotria subpunctata* Hiern
Candidatus Burkholderia subpunctata	BR-Lachenaud et al. 815	Cameroon	JN053622	JN054211	JN054082	*Psychotria subpunctata* Hiern
Candidatus Burkholderia trichardtensis	BR-Lemaire et al. 280	South Africa	JN053623	JN054212	JN054083	*Pavetta trichardtensis* Bremek.
Candidatus Burkholderia trichardtensis	BR-Lemaire et al. 282	South Africa	JN053624	JN054213	JN054084	*Pavetta trichardtensis* Bremek.
Candidatus Burkholderia trichardtensis	BR-Lemaire et al. 295	South Africa	JN053625	JN054214	JN054085	*Pavetta trichardtensis* Bremek.
Candidatus Burkholderia trichardtensis	BR-Lemaire et al. 299	South Africa	JN053626	-	JN054086	*Pavetta trichardtensis* Bremek.
Candidatus Burkholderia uapacifolia	BR-Dessein et al. 1732	Gabon	JN053627	JN054215	JN054087	*Psychotria uapacifolia* spec. nov.
Candidatus Burkholderia uapacifolia	BR-Dessein et al. 2084	Gabon	JN053628	JN054216	JN054088	*Psychotria uapacifolia* spec. nov.
Candidatus Burkholderia umbellifera	BR-Dessein et al. 2414	Gabon	JN053629	JN054217	JN054089	*Psychotria umbellifera* E.M.A.Petit
Candidatus Burkholderia vanwykii	BR-Lemaire et al. 154	South Africa	JN053630	-	JN054090	*Pavetta vanwykiana* Bridson
Candidatus Burkholderia vanwykii	BR-Lemaire et al. 168	South Africa	JN053631	-	JN054091	*Pavetta vanwykiana* Bridson
Candidatus Burkholderia vanwykii	BR-Lemaire et al. 181	South Africa	JN053632	-	JN054092	*Pavetta vanwykiana* Bridson
Candidatus Burkholderia verschuerenii	BR-without voucher	Unknown	JN053633	JN054218	JN054093	*Psychotria verschuerenii* De Wild.
Candidatus Burkholderia verschuerenii	BR-Dessein et al. 1760	Gabon	JN053634	JN054219	JN054094	*Psychotria verschuerenii* De Wild.
Candidatus Burkholderia verschuerenii	BR-750204	Cameroon	JN053635	JN054220	JN054095	*Psychotria verschuerenii* De Wild.
Candidatus Burkholderia verschuerenii	BR-Lachenaud et al. 655A	Cameroon	JN053636	JN054221	JN054096	*Psychotria verschuerenii* De Wild.
Candidatus Burkholderia verschuerenii	BR-Lachenaud et al. 655B	Cameroon	JN053637	JN054222	JN054097	*Psychotria verschuerenii* De Wild.
Candidatus Burkholderia virens	RBGE-20042025	China	JF416286	-	JF416292	*Ardisia virens* Kurz
