# Supplementary material for: Bacterial Leaf Symbiosis in Angiosperms: Host Specificity without Co-Speciation
Source: PLoS One. 2011 Sep 7;6(9):e24430. doi: 10.1371/journal.pone.0024430 (PMC3168474; doi:10.1371/journal.pone.0024430)
Supplement: Table S2 — Accession numbers, voucher data and origin of bacterial endosymbionts and host plants used in the co-speciation analysis. Specimens were obtained from the National Botanic Garden of Belgium (BR). - = not sequenced. (PDF) [file pone.0024430.s002.pdf]

		Accession numbers of host data								
**Taxon**	**Strain/Voucher**	***rps16***	***trnG***	***trnLF***	***petD***	***rps16-trnK***	***rpl32-trnL***	***petL-psbE***	***ndhF-rpl32***	***psbD-trnT***
Psychotria radicifera spec. nov.	BR-Dessein et al. 1769	JN053844	JN053896	JN053942	JN053772	JN053871	JN053922	JN053798	JN053747	JN053822
Psychotria anthocleistifolia spec. nov.	BR-Dessein et al. 1875	JN053845	JN053897	JN053943	JN053773	JN053872	JN053923	JN053799	JN053748	-
Psychotria brachyanthoides De Wild.	BR-2009044596	JN053846	JN053899	JN053944	JN053774	-	JN053924	JN053800	JN053749	JN053823
Psychotria brachyantha Hiern	BR-Lachenaud et al. 876B	JN053847	JN053898	JN053945	JN053775	JN053874	JN053925	JN053801	JN053750	-
Psychotria calva Hiern	BR-19620512	JN053848	JN053900	JN053947	JN053776	JN053875	JN053926	JN053802	JN053751	JN053824
Psychotria camerunensis E.M.A.Petit	BR-Dessein et al. 1465	JN053852	JN053904	JN053949	JN053779	JN053879	-	-	JN053753	JN053826
Psychotria expansissima K.Schum.	BR-Groeninckx et al. 4	JN053853	JN053905	-	JN053780	-	JN053928	JN053805	JN053755	JN053827
Psychotria fleuryana E.M.A.Petit	BR-Dessein et al. 2578	JN053851	JN053903	JN053948	JN053778	JN053878	JN053927	JN053804	JN053754	-
Psychotria humilis Hiern	BR-Dessein et al. 1497	JN053854	-	JN053950	JN053781	JN053880	JN053929	JN053806	JN053756	JN053828
Psychotria kikwitensis De Wild.	BR-Dessein et al. 1043	JN053855	JN053906	JN053951	JN053782	JN053881	JN053930	JN053807	JN053757	JN053829
Psychotria kimuenzae De Wild.	BR-Stoffelen et al. 7	JN053856	JN053907	JN053952	JN053783	JN053882	-	JN053808	JN053758	-
Psychotria kirkii Hiern	BR-2002152647	JN053857	JN053908	JN053953	JN053784	JN053883	JN053931	JN053809	JN053759	JN053830
Psychotria konguensis Hiern	BR-Dessein et al. 1705	JN053858	JN053909	JN053954	JN053785	JN053884	-	JN053810	-	JN053831
Psychotria letouzeyi E.M.A.Petit	BR-Dessein et al. 2140	JN053859	JN053910	JN053955	JN053786	JN053885	JN053932	JN053811	JN053760	JN053838
Psychotria mannii Hiern	BR-Dessein et al. 2299	JN053860	JN053911	JN053956	JN053787	JN053873	-	JN053812	JN053761	JN053832
Psychotria nigropunctata Hiern	BR-Stoffelen et al. 13	JN053861	JN053912	JN053957	JN053788	JN053886	JN053933	JN053813	JN053762	JN053833
Psychotria pendulothyrsa spec. nov.	BR-Dessein et al. 2438	JN053862	JN053913	JN053958	JN053789	JN053887	JN053934	-	JN053763	JN053834
Psychotria pumila Hiern	BR-2004143571	JN053864	JN053915	JN053960	JN053791	JN053889	JN053936	JN053815	JN053765	JN053836
Psychotria recurva Hiern	BR-Dessein et al. 2575	JN053850	JN053901	JN053946	JN053777	JN053876	-	-	JN053752	-
Psychotria rhizomatosa De Wild.	BR-Dessein et al. 2368	JN053865	JN053916	JN053961	JN053792	JN053890	JN053937	JN053816	JN053766	JN053837
Psychotria rubripilis K.Schum.	BR-Dessein et al. 1806	JN053866	JN053917	JN053962	JN053793	JN053891	JN053938	JN053817	JN053767	JN053839
Psychotria rubristipulata R.D.Good	BR-Dessein et al. 2107	JN053867	JN053918	JN053963	JN053794	JN053892	JN053939	JN053818	JN053768	JN053840
Psychotria spithamea S.Moore	BR-2000194762	JN053863	JN053914	JN053959	JN053790	JN053888	JN053935	JN053814	JN053764	JN053835
Psychotria subpunctata Hiern	BR-Dessein et al. 2475	JN053849	JN053902	-	-	JN053877	-	JN053803	-	JN053825
Psychotria uapacifolia spec. nov.	BR-Dessein et al. 2084	JN053868	JN053919	JN053964	JN053795	JN053893	JN053940	JN053819	JN053769	JN053841
Psychotria umbellifera E.M.A.Petit	BR-Dessein et al. 2414	JN053869	JN053920	JN053965	JN053796	JN053894	-	JN053820	JN053770	JN053842
Psychotria verschuerenii De Wild.	BR-Dessein et al. 1760	JN053870	JN053921	JN053966	JN053797	JN053895	JN053941	JN053821	JN053771	JN053843

Accession numbers of endosymbiont data				
**Taxon**	**16S rDNA**	***recA***	***gyrB* (part 1)**	***gyrB* (part 2)**
Burkholderia multivorans	AB092606	AF143775	DQ124425	DQ124425
Candidatus Burkholderia anthocleistifolia	JN053517	JN054101	JN053970	JN053723
Candidatus Burkholderia brachyanthoides	JN053524	JN054108	JN053977	JN053724
Candidatus Burkholderia brachyantha	JN053526	JN054110	JN053979	JN053745
Candidatus Burkholderia calva	HQ849116	HQ849172	JF295009	JN053725
Candidatus Burkholderia cameruensis	JN053530	JN054114	JN053983	JN053727
Candidatus Burkholderia expanssisima	JN053548	JN054128	JN054000	JN053728
Candidatus Burkholderia fleuryana	JN053551	JN054130	JN054003	JN053746
Candidatus Burkholderia humilis	JN053556	JN054136	JN054010	JN053729
Candidatus Burkholderia kikwitensis	JN053560	JN054141	JN054015	-
Candidatus Burkholderia kimuenzae	JN053561	JN054142	JN054016	JN053730
Candidatus Burkholderia kirkii	HQ849111	HQ849167	HQ849222	-
Candidatus Burkholderia konguensis	JN053565	JN054150	JN054024	JN053731
Candidatus Burkholderia letouzeyi	JN053580	JN054166	JN054039	JN053732
Candidatus Burkholderia mannii	JN053587	JN054176	JN054047	JN053733
Candidatus Burkholderia nigropunctata	HQ849118	HQ849174	HQ849228	JN053734
Candidatus Burkholderia pendulothyrsa	JN053589	JN054179	JN054050	JN053735
Candidatus Burkholderia pumila	JN053591	JN054181	JN054052	JN053737
Candidatus Burkholderia recurva	JN053594	JN054184	JN054055	JN053726
Candidatus Burkholderia rhizomatosa	JN053597	JN054186	JN054061	JN053738
Candidatus Burkholderia rubripilis	JN053605	-	JN054065	JN053739
Candidatus Burkholderia rubristipulata	JN053610	-	JN054070	JN053740
Candidatus Burkholderia spithamea	JN053620	-	JN054079	JN053736
Candidatus Burkholderia subpunctata	-	JN054209	JN054080	JN053741
Candidatus Burkholderia uapacifolia	JN053628	JN054216	JN054088	JN053742
Candidatus Burkholderia umbellifera	JN053629	JN054217	JN054089	JN053743
Candidatus Burkholderia verschuerenii	JN053634	JN054219	JN054094	JN053744
