# Supplementary material for: Bacterial Leaf Symbiosis in Angiosperms: Host Specificity without Co-Speciation
Source: PLoS One. 2011 Sep 7;6(9):e24430. doi: 10.1371/journal.pone.0024430 (PMC3168474; doi:10.1371/journal.pone.0024430)
Supplement: Table S3 — Accession numbers and voucher data of nodulated genera used in the age estimation analysis of leaf nodulated Rubiaceae. Specimens were obtained from the National Botanic Garden of Belgium (BR), the Royal Botanic Garden of Edinburgh (RBGE), the Gothenburg herbarium (GB), the herbarium of Leiden (L) and the herbarium of the Missouri Botanical Garden (MO). - = not sequenced. (PDF) [file pone.0024430.s003.pdf]

Group	Taxon	Voucher	Accession numbers			
			petD	***trnLF***	***trnG***	***rps16***
Cinchonoideae						
Ixoridinae	*Coffea stenophylla* G.Don	BR-19370053	JF916975	JF916964	JF916953	JF916942
	Empogona kirkii Hook.f.	BR-19761052	JF916976	JF916965	JF916954	JF916943
	Sericanthe andongensis (Hiern) Robbr.	BR-Dessein et al. 1097	JF916977	JF916966	JF916955	JF916944
	Sericanthe andongensis (Hiern) Robbr.	BR-Chapman 6150	JF916978	JF916967	JF916956	JF916945
	Sericanthe auriculata (Keay) Robbr.	BR-Dessein et al. 1467	JF916979	JF916968	JF916957	JF916946
	Sericanthe auriculata (Keay) Robbr.	BR-Dessein et al. 1516	JF916980	JF916969	JF916958	JF916947
	Sericanthe odoratissima (K.Schum.) Robbr.	BR-Polhill et al. 5007A	-	JF916970	JF916959	JF916948
	Sericanthe odoratissima (K.Schum.) Robbr.	BR-Salubeni 3135	JF916981	JF916971	JF916960	JF916949
	Sericanthe aff. petitii (N.Hallé) Robbr.	BR-Dessein et al. 1512	JF916982	JF916972	JF916961	JF916950
	Sericanthe aff. petitii (N.Hallé) Robbr.	BR-Lachenaud et al. 658	JF916983	JF916973	JF916962	JF916951
	Sericanthe spec. nov.	BR-Dessein et al. 2608	JF916984	JF916974	JF916963	JF916952
	Pavetta batesiana Bremek.	BR-Dessein et al. 2071	JN054234	-	JN053667	JN053699
	Pavetta batesiana Bremek.	BR-Dessein et al. 1984	JN054235	JN053639	JN053668	JN053701
	Pavetta bidentata Hiern	BR-Lachenaud et al. 593	JN054223	JN053644	JN053676	JN053706
	Pavetta hispida Hiern	BR-Dessein et al. 1943	JN054230	JN053638	JN053669	JN053700
	Pavetta hispida Hiern	BR-Dessein et al. 3176	JN054229	JN053643	JN053670	JN053705
	Pavetta hookeriana Hiern	BR-Dessein et al. 3046	JN054227	JN053640	JN053671	JN053702
	Pavetta hookeriana Hiern	BR-Dessein et al. 3048	JN054228	JN053641	JN053672	JN053703
	Pavetta rigida Hiern	BR-Lachenaud et al. 877	JN054225	-	JN053675	JN053708
	Pavetta rigida Hiern	BR-Lachenaud et al. 694	JN054224	JN053645	JN053674	JN053707
	Pavetta rigida Hiern	BR-Dessein et al. 3167	JN054226	JN053642	JN053673	JN053704
	Pavetta schumanniana F.Hoffm. ex K.Schum.	BR-Dessein et al. 1099	JN054231	JN053646	JN053677	JN053709
	Pavetta schumanniana F.Hoffm. ex K.Schum.	BR-2001944257	JN054232	JN053647	JN053678	JN053710
	Pavetta schumanniana F.Hoffm. ex K.Schum.	BR-2004143066	JN054233	-	JN053679	JN053711
Rubioideae						
Psychotriidinae	*Psychotria chrysantha* Merr. & L.M.Perry	L-Pullen 381	JN054238	JN053651	JN053683	AF410704
	Psychotria lucens Hiern	BR-16620513	JN054239	JN053652	JN053684	AF410733
	Psychotria peduncularis (Salisb.) Steyerm.	BR-073791	JN054240	JN053654	JN053686	AF410742
	Psychotria nervosa Benth.	GB-Rova et al. 2249	JN054241	JN053653	JN053685	AF410738
	Psychotria anceps Kunth	MO-Taylor 12078	JN054242	JN053650	JN053682	AF147544
	Myrmecodia horrida C.R.Huxley & Jebb	without voucher	JN054236	JN053649	JN053681	AF410690
	Psychotria psychotrioides (Helier) Fosberg	BR-Dessein et al. 2926	JN054253	JN053655	JN053687	JN053721
	Psychotria anetoclada Hiern	BR-Dessein et al. 3138	JN054252	JN053656	JN053688	JN053722
	Myrmecodia tuberosa Jack	GB-Andersson 2217	JN054237	JN053648	JN053680	AF000950
	Psychotria calva Hiern	BR-19620512	JN054243	JN053666	JN053698	JN053720
	Psychotria calva Hiern	BR-626512	JN054244	JN053665	JN053697	AF410702
	Psychotria kirkii Hiern	BR-1998182519	JN054246	JN053662	JN053694	JN053717
	Psychotria kirkii Hiern	BR-2002152647	JN054247	JN053661	JN053693	JN053716

	Psychotria rhizomatosa De Wild.	BR-Dessein et al. 2368	JN054248	JN053660	JN053692	JN053715
	Psychotria rhizomatosa De Wild.	BR-Dessein et al. 2099	JN054249	JN053659	JN053691	JN053714
	Psychotria rubripilis K.Schum.	BR-Dessein et al. 1806	JN054250	JN053658	JN053690	JN053713
	Psychotria rubripilis K.Schum.	BR-Dessein et al. 1973	JN054251	JN053657	JN053689	JN053712
	Psychotria kikwitensis De Wild.	BR-Dessein et al. 1043	JN054245	JN053663	JN053695	JN053718
	Psychotria kikwitensis De Wild.	BR-2004145187	-	JN053664	JN053696	JN053719
