# Supplementary material for: Bacterial Leaf Symbiosis in Angiosperms: Host Specificity without Co-Speciation
Source: PLoS One. 2011 Sep 7;6(9):e24430. doi: 10.1371/journal.pone.0024430 (PMC3168474; doi:10.1371/journal.pone.0024430)
Supplement: Table S4 — Accession numbers and voucher data of nodulated Ardisia used in the age estimation analysis of leaf nodulated Primulaceae. Specimens were obtained from the National Botanic Garden of Belgium (BR) and the Royal Botanic Garden of Edinburgh (RBGE). - = not sequenced. (PDF) [file pone.0024430.s004.pdf]

Family	Taxa	Voucher	Accession numbers		
			matK	*rps16*	*trnLF*
Primulaceae					
	Ardisia guianensis (Aubl.) Mez	BR-19762361	JF416269	JF416253	JF416263
	Ardisia sanguinolenta Blume	BR-19581069	JF416270	JF416256	JF416267
	Ardisia polycephala Wight	RBGE-19671776	JF416277	JF416254	JF416264
	Ardisia japonica Blume	BR-19812125	JF416274	JF416248	JF416259
	Ardisia humilis Vahl	BR-19391577	JF416272	JF416257	JF416268
	Ardisia involucrata Kurz	RBGE-19715897	JF416273	-	JF416266
	Ardisia solanacea Roxb.	RBGE-20011041	JF416278	JF416255	JF416265
	Ardisia opegrapha Oerst.	RBGE-19696189	JF416276	JF416249	JF416260
	Ardisia crenata Sims.	BR-19073685	JF416271	JF416252	JF416262
	Ardisia virens Kurz	RBGE-20042025	JF416279	JF416251	-
	Ardisia mamillata Hance	BR-10005023	JF416275	JF416250	JF416261
	Rapanea guianensis Aubl.	BR-19810166	JF416280	JF416247	JF416258
