# Supplementary material for: Bacterial Leaf Symbiosis in Angiosperms: Host Specificity without Co-Speciation
Source: PLoS One. 2011 Sep 7;6(9):e24430. doi: 10.1371/journal.pone.0024430 (PMC3168474; doi:10.1371/journal.pone.0024430)
Supplement: Table S5 — DNA sequences for primers used in this study. References (i.e. [17], [77]–[84]) are provided of previously published sequence primers. (PDF) [file pone.0024430.s005.pdf]

Region	Primer	Primer sequence (5'-3')	Reference
**Endosymbiont; *Burkholderia***			
16S rDNA	16SB (forward)	AGAGTTTGATCCTGGCTCAG	Van Oevelen et al. 2001
	16SE (reverse)	AAGGAGGTGATCCAGCCGCA	Van Oevelen et al. 2001
	16S2 (reverse)	CAGACTGCGATCCGGACTACGATC	Lemaire et al. 2010
recA	*recA*-F (forward)	AGGACGATTCATGGAAGAWAGC	Spilker et al. 2009
	recA-R (reverse)	GACGCACYGAYGMRTAGAACTT	Spilker et al. 2009
gyrB	*gyrB*-F (forward)	ACCGGTCTGCAYCACCTCGT	Spilker et al. 2009
	gyrB-R (reverse)	YTCGTTGWARCTGTCGTTCCACTGC	Spilker et al. 2009
	PC3 (forward)	CGGATCCATSGTSGTTTCC	Tabacchioni et al. 2008
	PC9r (reverse)	GAAGTSGCGATGCAGTGGAACGA	Tabacchioni et al. 2008
**Host plant; *Pavetta, Psychotria and Sericanthe***			
rps16	*rps16*-F (forward	GTGGTAGAAAGCAACGTGCGACTT	Oxelman et al. 1997
	rps16-R2 (reverse)	TCGGGATCGAACATCAATTGCAAC	Oxelman et al. 1997
trnG	*trnG*-1F (forward)	TAGCGGGTATAGTTTAGTGG	Tesfaye et al. 2007
	trnG-725R (reverse)	ATCGTTAGCTTGGAAGGCT	Tesfaye et al. 2007
trnL-trnF	*trnL*-c (forward)	CGAAATCGGTAGACGCTACG	Taberlet et al. 1991
	trnL-f (reverse)	ATTTGAACTGGTGACACGAG	Taberlet et al. 1991
petD	*petD*-1365 (forward)	TTGACYCGTTTTTATAGTTTAC	Löhne & Borsch 2004
	petD-738 (reverse)	AATTTAGCYCTTAATACAGG	Löhne & Borsch 2004
rps16-trnK	*rps16xF* (forward)	TCTACCGCTTTCTAGTTATCATCCC	Shaw et al. 2007
	trnK (reverse)	TTA AAAGCCGAGTACTCTACC	Shaw et al. 2007
rpl32-trnL	*rpl32-F* (forward)	CAGTTCCAAAAAAACGTACTTC	Shaw et al. 2007
	trnL (reverse)	CTGCTTCCTAAGAGCAGCGT	Shaw et al. 2007
ndhF-rpl32	ndhF (forward)	GAAAGGTATKATCCAYGMATATT	Shaw et al. 2007
	rpL32-R (reverse)	CCAATATCCCTTYYTTTTCCAA	Shaw et al. 2007
psbD-trnT	*psbD* (forward)	CTCCGTARCCAGTCATCCATA	Shaw et al. 2007
	trnT-R (reverse)	CCCTTTTAACTCAGTGGTAG	Shaw et al. 2007
petL-psbE	*petL* (forward)	AGTAGAAAACCGAAATAACTAGTTA	Shaw et al. 2007
	psbE (reverse)	TATCGAATACTGGTAATAATATCAGC	Shaw et al. 2007
**Host plant; *Ardisia***			
rps16	*rps16*_2F (forward	GAA GGA CAC GAT CCG TTG TGG AT	Bremer et al. 2002 (adapted)
	*rps16*_R3 (reverse)	CGA TAG ACG GCT CAT TGG GAT A	Bremer et al. 2002
trnL-trnF	*trnT-F*_cF (forward)	CGAAATCGGTAGACGCTACG	Taberlet et al. 1991
	*trnT-F*_fR (reverse)	ATTTGAACTGGTGACACGAG	Taberlet et al. 1991 (adapted)
matK	*matK*_1Fb (forward)	TATATCCGCTTATATTTCAGGAGT	Bremer et al. 2002 (adapted)
	*matK*_5F (forward)	GGTACGGAATCAAATGCTAGAAAA T	Bremer et al. 2002 (adapted)
	*matK*_1R (reverse)	GAACTAGTCGGATGGAGTAG	Bremer et al. 2002
	*matK*_3R (reverse)	GATCCGCTATAATAATGAGA	Bremer et al. 2002 (adapted)
