# Supplementary material for: Bacterial Leaf Symbiosis in Angiosperms: Host Specificity without Co-Speciation
Source: PLoS One. 2011 Sep 7;6(9):e24430. doi: 10.1371/journal.pone.0024430 (PMC3168474; doi:10.1371/journal.pone.0024430)
Supplement: Table S6 — 16S rRNA accession numbers, voucher data and origin of endosymbionts of leaf nodulated angiosperms, gut symbionts of stinkbugs and environmental isolates. Specimens were obtained from the National Botanic Garden of Belgium (BR) and the Royal Botanic Garden of Edinburgh (RBGE). (PDF) [file pone.0024430.s006.pdf]

Origin	Taxon	Voucher	Accession numbers
Stinkbugs	Endosymbiont of *Yemma exilis*		AB558174
	Endosymbiont of *Yemma exilis*		AB558175
	Endosymbiont of *Yemma exilis*		AB558176
	Endosymbiont of *Dimorphopterus pallipes*		AB558177
	Endosymbiont of *Dimorphopterus pallipes*		AB558178
	Endosymbiont of *Dimorphopterus pallipes*		AB558179
	Endosymbiont of *Paromius exiguous*		AB558180
	Endosymbiont of *Paromius exiguous*		AB558181
	Endosymbiont of *Paromius exiguous*		AB558182
	Endosymbiont of *Togo hemipterus*		AB558183
	Endosymbiont of *Togo hemipterus*		AB558184
	Endosymbiont of *Panaorus japonicus*		AB558185
	Endosymbiont of *Pachygrontha antennata*		AB558186
	Endosymbiont of *Pachygrontha antennata*		AB558187
	Endosymbiont of *Pachygrontha antennata*		AB558188
	Endosymbiont of *Pachygrontha antennata*		AB558189
	Endosymbiont of *Pachygrontha antennata*		AB558190
	Endosymbiont of *Molipteryx fuliginosa*		AB558191
	Endosymbiont of *Acanthocoris sordidus*		AB558192
	Endosymbiont of *Acanthocoris sordidus*		AB558193
	Endosymbiont of *Acanthocoris sordidus*		AB558194
	Endosymbiont of *Notobitus meleagris*		AB558195
	Endosymbiont of *Hygia opaca*		AB558196
	Endosymbiont of *Hygia lativentris*		AB558197
	Endosymbiont of *Hygia lativentris*		AB558198
	Endosymbiont of *Hygia lativentris*		AB558199
	Endosymbiont of *Hygia lativentris*		AB558200
	Endosymbiont of *Homoeoceus dilatatus*		AB558201
	Endosymbiont of *Homoeocerus unipunctatus*		AB558202
	Endosymbiont of *Plinachtus bicoloripes*		AB558203
	Endosymbiont of *Cletus trigonus*		AB558204
	Endosymbiont of *Cletus punctiger*		AB558205
	Endosymbiont of *Cletus punctiger*		AB558206
	Endosymbiont of *Cletus rusticus*		AB558207
	Endosymbiont of *Riptortus pedestris*		AB558208
	Endosymbiont of *Riptortus pedestris*		AB558209
	Endosymbiont of *Riptortus pedestris*		AB558210
	Endosymbiont of *Riptortus pedestris*		AB558211
	Endosymbiont of *Riptortus linearis*		AB558212
	Endosymbiont of *Leptocorisa chinensis*		AB558213
	Endosymbiont of *Leptocorisa chinensis*		AB558214
	Endosymbiont of *Leptocorisa chinensis*		AB558215
	Endosymbiont of *Daclera levana*		AB558216
Environmental samples	*Burkholderia sp.* WD263		AJ292641
	Burkholderia sp. WD2116		AJ292648
	Burkholderia sp. WD206		AJ292638
	Burkholderia sp. TFA2		AB232338
	Burkholderia sp. TFA1		AB232337
	Burkholderia sp. SFA4		AB232336
	Burkholderia sp. SJ98		DQ986324
	Burkholderia sp. SFA1		AB232333
	Burkholderia sp. S4.9		AF247496
	Burkholderia sp. PAMU-2.6		AB118226
	Burkholderia sp. OP-1		HM802212
	Burkholderia sp. NK8		AB208548
	Burkholderia sp. NF100		AB025790
	Burkholderia sp. N2P6		U37343
	Burkholderia sp. KU-25		AB266607
	Burkholderia sp. KFA2		AB232331
	Burkholderia sp. IFA2		AB232326
	Burkholderia sp. FDS-1		AY550913
	Burkholderia sp. AK-5		AB103080
	Burkholderia sp. NF23		AJ300698

Marine metagenome	*Burkholderia sp.* 408172		AACY020561965
Burkholderia	*Burkholderia glathei* LMG 14190		Y17052
	Burkholderia cepacia LMG 1222		EU024171
	Burkholderia vietnamiensis LMG 10929		AF097534
	Burkholderia stabilis LMG 14294		EU024183
	Burkholderia ambifaria MC40-6		NC010552
	Burkholderia gladioli LMG 2216		EU024168
	Burkholderia plantarii LMG 9035		U96933
Nodulated	*Candidatus* Burkholderia alatipes	BR-Dessein et al. 2547	JN053515
Endosymbionts	*Candidatus* Burkholderia andongensis	BR-Dessein et al. 1097	JF916921
	Candidatus Burkholderia anthocleistifolia	BR-Dessein et al. 1875	JN053517
	Candidatus Burkholderia bidentata	BR-Lachenaud et al. 593	JN053519
	Candidatus Burkholderia bifaria	BR-Dessein et al. 2862A	JN053520
	Candidatus Burkholderia brachyanthoides	BR-2009044596	JN053524
	Candidatus Burkholderia brachyantha	BR-Dessein et al. 2731	JN053525
	Candidatus Burkholderia brevipaniculata	BR-Dessein et al. 2916	JN053527
	Candidatus Burkholderia calva	BR-1962 0512	HQ849116
	Candidatus Burkholderia camerunensis	BR-Lachenaud et al. 862	JN053536
	Candidatus Burkholderia camerunensis	BR-Lachenaud et al. 717B	JN053535
	Candidatus Burkholderia catophylla	BR-Lemaire et al. 219	JN053540
	Candidatus Burkholderia cooperi	BR-Lemaire et al. 75	JN053541
	Candidatus Burkholderia crenata	RBGE-19696187	JF416283
	Candidatus Burkholderia darwiniana	BR-Dessein et al. 2682	JN053543
	Candidatus Burkholderia edentula	BR-Lemaire et al. 135	JN053547
	Candidatus Burkholderia expansissima	BR-Groeninckx et al. 4	JN053548
	Candidatus Burkholderia eylesii	BR-Lemaire et al. 253C	JN053550
	Candidatus Burkholderia fleuryana	BR-Dessein et al. 2675	JN053552
	Candidatus Burkholderia sp.	BR-Lachenaud et al. 882	JN053619
	Candidatus Burkholderia gardeniifolia	BR-Lemaire et al. 136	JN053555
	Candidatus Burkholderia hispidae	BR-Lachenaud et al. 732	HQ849123
	Candidatus Burkholderia humilis	BR-Dessein et al. 3175	JN053557
	Candidatus Burkholderia inandensis	BR-Lemaire et al. 244	JN053558
	Candidatus Burkholderia kikwitensis	BR-Dessein et al. 1043	JN053560
	Candidatus Burkholderia kimuenzae	BR-Stoffelen et al. 7	JN053561
	Candidatus Burkholderia kirkii	BR-1953 6779	HQ849109
	Candidatus Burkholderia konguensis	BR-Lachenaud et al. 932	JN053567
	Candidatus Burkholderia kotzei	BR-Lemaire et al. 126	JN053568
	Candidatus Burkholderia lanceolata	BR-Lemaire et al. 41	JN053570
	Candidatus Burkholderia leptophylla	BR-Lachenaud et al. 864	JN053578
	Candidatus Burkholderia letouzeyi	BR-Lachenaud et al. 931	JN053581
	Candidatus Burkholderia lokohensis	BR-Tosh et al. 238	JN053582
	Candidatus Burkholderia mamillata	BR-10005024	JF416285
	Candidatus Burkholderia mannii	BR-Dessein et al. 2493	JN053583
	Candidatus Burkholderia nigropunctata	BR-Stoffelen et al. 13	HQ849119
	Candidatus Burkholderia pendulothyrsa	BR-Dessein et al. 2438	JN053589
	Candidatus Burkholderia petitii	BR-Lachenaud et al. 658	JF916922
	Candidatus Burkholderia pumila	BR-2004 1435-71	JN053591
	Candidatus Burkholderia kirkii	BR-De Block et al. 372	JN053592
	Candidatus Burkholderia recurva	BR-Dessein et al. 2575	JN053594
	Candidatus Burkholderia rhizomatosa	BR-Dessein et al. 1772	JN053596
	Candidatus Burkholderia rigidae	BR-Lachenaud et al. 877	HQ849121
	Candidatus Burkholderia rubripilis	BR-Dessein et al. 3174	JN053609
	Candidatus Burkholderia rubristipulata	BR-Dessein et al. 2107	JN053610
	Candidatus Burkholderia schumannianae	BR-Lemaire et al. 99	HQ849129
	Candidatus Burkholderia trichardtensis	BR-Lemaire et al. 299	JN053626
	Candidatus Burkholderia uapacifolia	BR-Dessein et al. 2084	JN053628
	Candidatus Burkholderia umbellifera	BR-Dessein et al. 2414	JN053629
	Candidatus Burkholderia vanwykii	BR-Lemaire et al. 181	JN053632
	Candidatus Burkholderia verschuerenii	BR-Lachenaud et al. 655B	JN053637
	Candidatus Burkholderia virens	RBGE-20042025	JF416286
outgroup	*Ralstonia picketii* 12J		NC010682
