# Supplementary figures and images for: Bacterial Leaf Symbiosis in Angiosperms: Host Specificity without Co-Speciation
Source: PLoS One. 2011 Sep 7;6(9):e24430. doi: 10.1371/journal.pone.0024430 (PMC3168474; doi:10.1371/journal.pone.0024430)

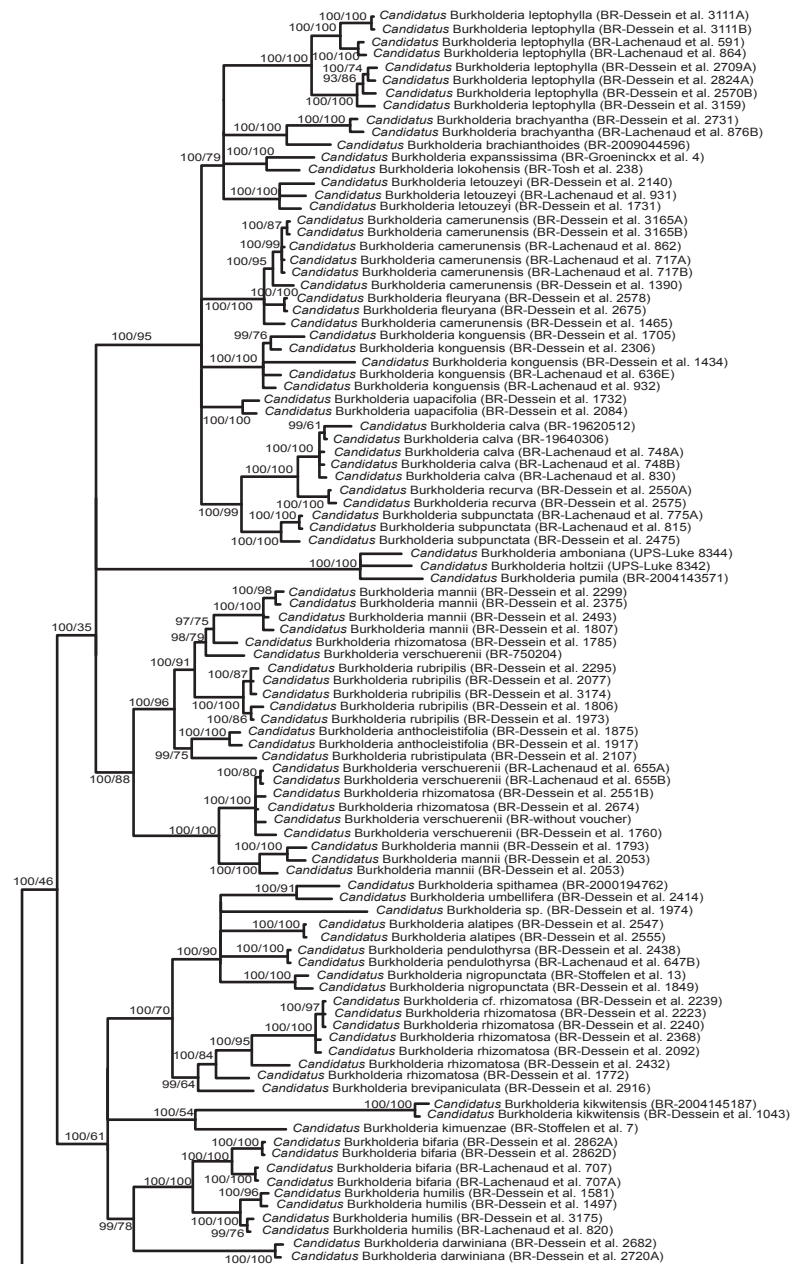

Nodulating endosymbionts  
Non-nodulating *Burkholderia*

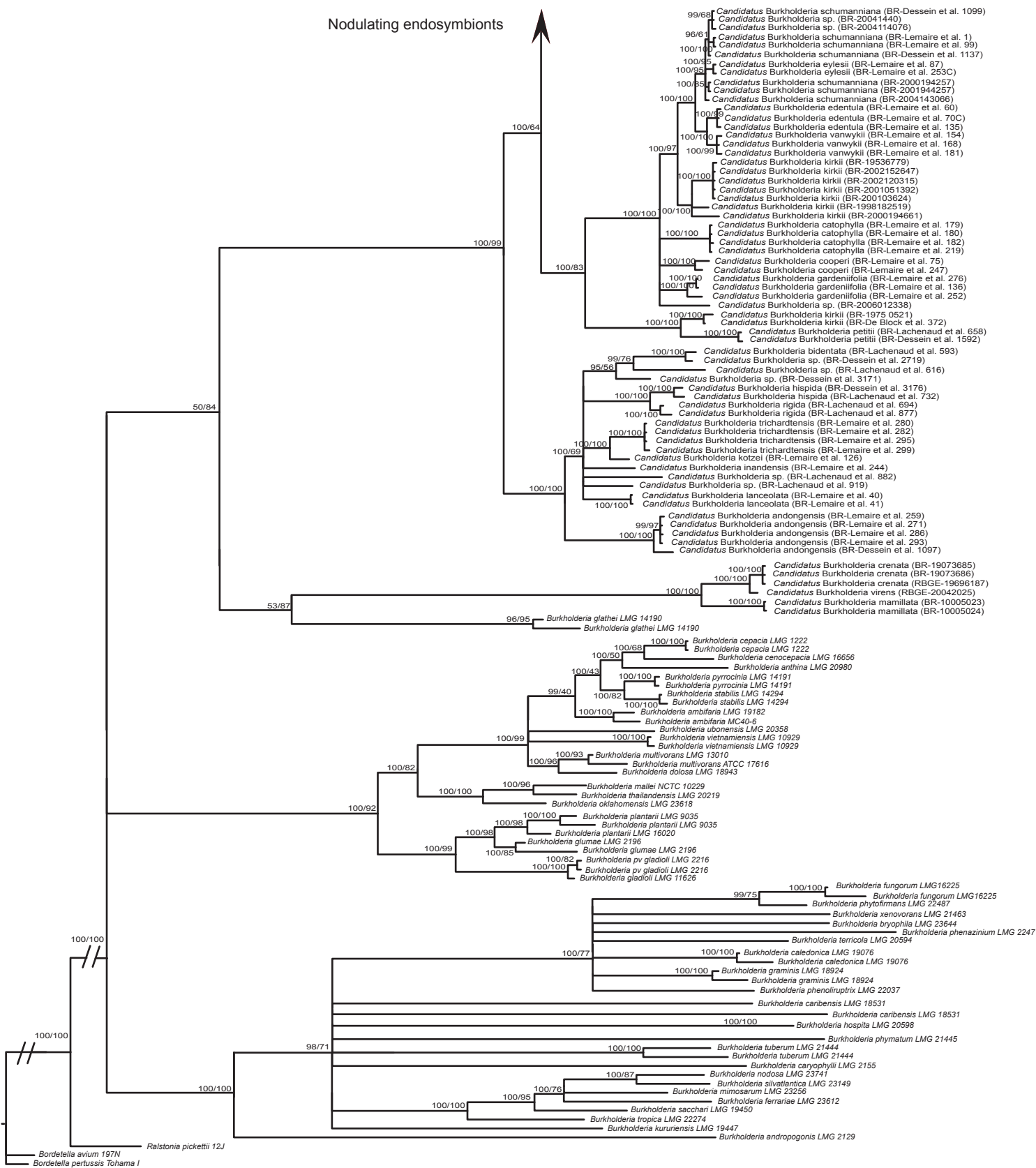

0.2

Supplement: Figure S1 — Phylogenetic relationships within leaf nodulated Burkholderia species based on phylogenetic analysis of 16S rRNA, recA and gyrB data. Support values of Bayesian and Maximum Likelihood analyses are given at the nodes (Bayesian posterior probabilities - bootstrap values from the Maximum Likelihood analysis). The scale bar represents 0.2 substitutions per site. (PDF) [file pone.0024430.s007.pdf]

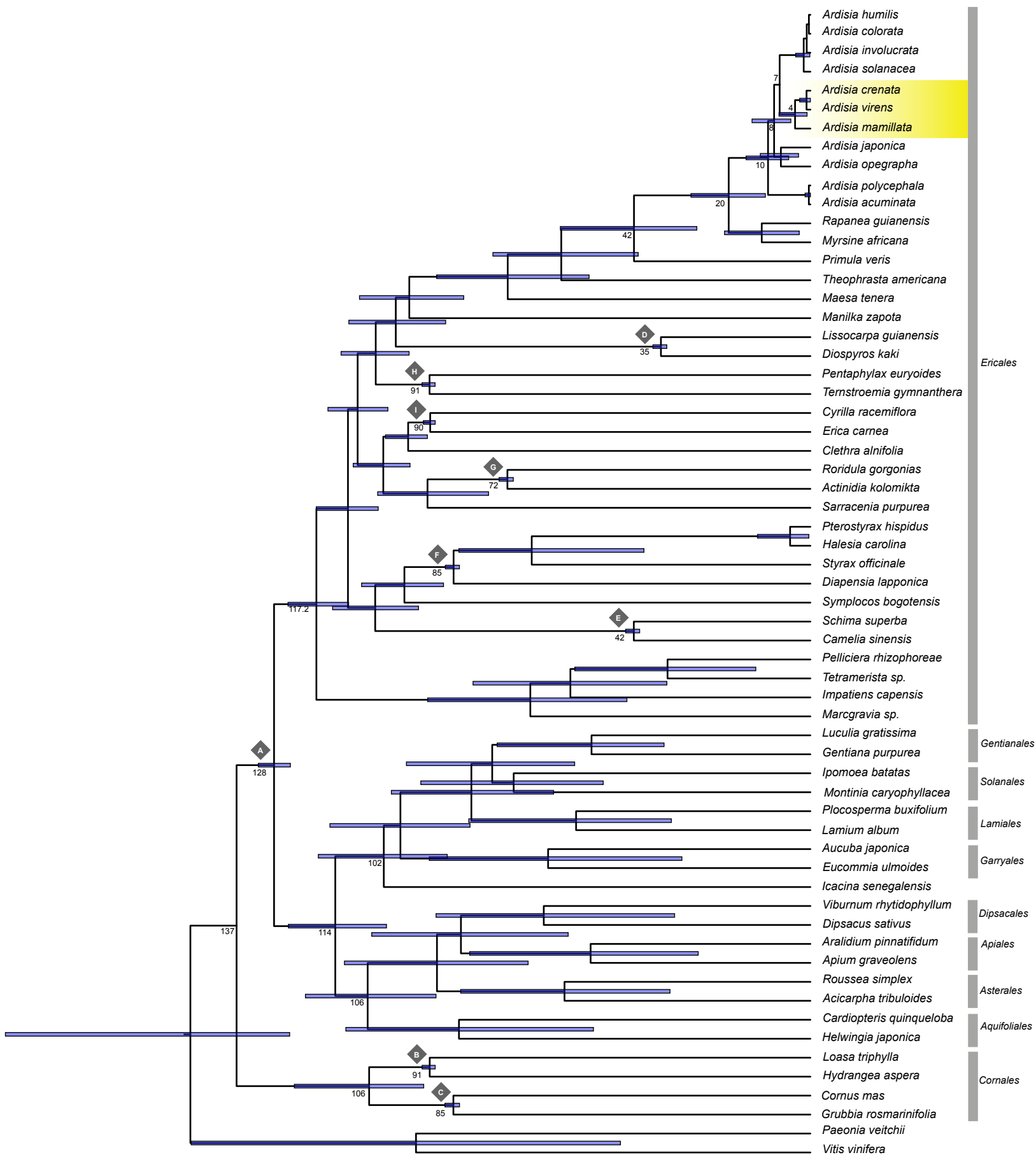

Supplement: Figure S2 — Phylogenetic chronogram of Rubiaceae based on rps16 , trnLF , trnG and petD sequence data obtained with a Bayesian relaxed clock analysis. Bars illustrate the 95% posterior probability intervals on age estimates. Numbers within black boxes indicate calibrated nodes. Yellow shading denotes leaf nodulated lineages. Scale bar below tree measure Mya. (PDF) [file pone.0024430.s008.pdf]

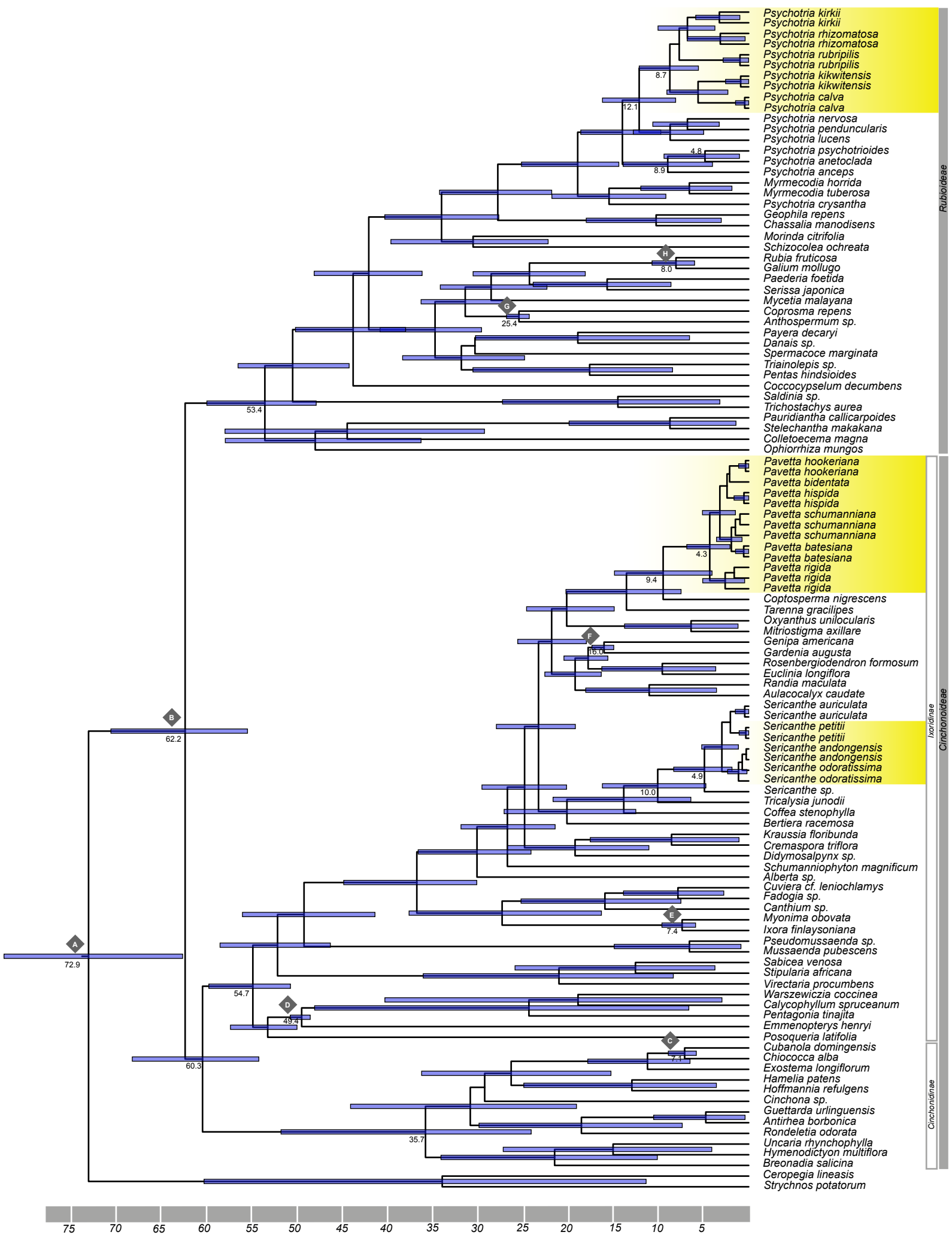

Supplement: Figure S3 — Phylogenetic chronogram of asterids based on matK , trnLF and rps16 sequence data obtained with a Bayesian relaxed clock analysis. Bars illustrate the 95% posterior probability intervals on age estimates. Numbers within black boxes indicate calibrated nodes. Yellow shading denotes leaf nodulated lineages. Scale bar below tree measure Mya. (PDF) [file pone.0024430.s009.pdf]
